# Supporters of Germany’s far-right AfD party are concerned about immigration rather than feeling deprived

**DOI:** 10.1371/journal.pone.0350403

**Published:** 2026-07-21

**Authors:** Martin Schröder, Moritz Rehm, Anja Röcke

**Affiliations:** 1 Department of European Social Research, Saarland University, Saarbrücken, Germany; 2 Department of European Social Research, Saarland University and Sciences Po Paris (Center for European Studies and Comparative Politics), Saarbrücken, Germany; Aix-Marseille Universite, FRANCE

## Abstract

Is concern about immigration sufficient to account for the increasing success of Germany’s right-wing populist party Alternative für Deutschland (AfD)? Or does socio-economic deprivation also matter? Using German Socio-Economic Panel (SOEP) data from 2014 to 2024, we find that concern about immigration alone accounts for about 75 percent of explicable variation in AfD support and even for 90 percent of explicable variation in voting behaviour. Therefore, variables that measure deprivation explain comparatively little additional variation in AfD support and even less variation in voting for the AfD. Previous research has shown that immigration-concern predicts AfD support. Our results extend this by arguing that the direct relationship between immigration-concern and AfD support is so strong that deprivation variables neither seem to have much additional explanatory power, nor strongly confound the direct relationship between immigration-concern and AfD support. These results undermine the much-cited “losers of modernisation hypothesis”, not only in the sense that socio-economic conditions may be a weaker explanation than cultural ones, but even by questioning whether socio-economic explanations of AfD support have much additional value over cultural explanations at all.

## 1. Introduction

The Alternative für Deutschland (AfD) has turned from a conservative ordoliberal Euro-sceptic movement into a populist far-right people’s party, which secured representation in 14 of Germany’s 16 regional parliaments and has become the second-strongest party in Germany’s federal Bundestag. Some surveys even see the AfD as Germany’s strongest party, receiving 26 per cent of votes [Forsa survey 12.08.2024; 1–3].

Which constituents stand behind this remarkable success? The typical far-right Western European party supporter is often portrayed as a lower-educated blue-collar worker who feels left behind by socio-economic modernization [4,5]. AfD supporters are described similarly [6–10]. Yet what distinguishes the AfD from other parties is not any type of policy that would help this constituency. Instead, the AfD sets itself apart through its populist radical-right anti-immigration program [11], which should make the party particularly appealing to those who are concerned about immigration [12, also cf. 13,14–18]. Indeed, studies find that AfD supporters are critical of immigration [12,13,19–22]. This seems to be particularly the case since the mid-2010s, when an influx of immigrants and a souring of opinion on immigration in Germany is said to have increased AfD support [12,13,23].

Contrary to a y-centred approach (explaining AfD support), we contribute to the debate on whether socio-economic deprivation or fear of immigration explains far-right party support by adopting an x-centred approach, which investigates to which degree anti-immigration attitudes alone can explain AfD support sufficiently. While the connection between immigration concern and AfD support has been widely discussed in the literature, several hypotheses around this relationship remain to be tested. First, only Hansen and Olsen (24) estimate the effect size of immigration concern on AfD support; and while they show a sizable effect, notably that the probability to vote for the AfD increases from 0 to about 30 per cent when moving from the highest versus lowest anti-immigrant sentiment, this leaves unclear to which degree concern about immigration alone is already a sufficient explanation for AfD support [7,24–26]. While this question is empirically not answered, there are several reasons to assume that concern about immigration may suffice to explain why individuals support the AfD, leaving little room for other explanations such as feeling deprived.

Firstly, the AfD is an economically liberal party and therefore offers little to those who feel deprived [26]. The AfD does not even try to gain support from economically deprived individuals, as it tends to downplay distributional conflicts in favour of cultural conflicts, mainly by trying to appeal to those who oppose immigration [9]. Moral Foundations Theory provides a mechanism that could explain why individuals support the AfD irrespective of their economic interest. It argues that individuals on the right of the political spectrum subscribe to a morality that draws sharp distinctions between in- and outgroups [27–30]. If this is indeed the case, then concern about immigration might not only be the primary, but virtually the sole reason, why individuals feel drawn to the AfD. This leads to our first hypotheses.

**Hypothesis 1a**: Compared to explaining AfD support with concern about immigration alone, adding variables that measure deprivation explains little additional variation.

**Hypothesis 1b**: The magnitude and direction of the relationship between AfD support and concern about immigration does not change substantially, irrespective of variables that measure deprivation.

Hypothesis 1a therefore suggests that if one knows whether an individual is concerned about immigration, little is gained by additionally knowing whether someone feels economically deprived. Hypothesis 1b suggests that the direct link between concern about immigration and AfD support is not due to material deprivation. Simplifying, one could say that Hypothesis 1a posits that other variables do not explain AfD support *in addition to* what concern about immigration explains, while Hypothesis 1b posits that other variables do not explain AfD support *instead of* what concern about immigration explains.

That concern about immigration is a sufficient explanation of AfD support is contradicted by the so-called ‘losers of modernisation’ hypothesis, which draws on earlier research on European far-right parties [4,5] to argue that deprivation is either an additional explanation for AfD support, an antecedent to concern about immigration, a mediator between immigration concern and AfD support or confounds the relationship. The basic theoretical argument is that individuals with low economic and human capital turn to far-right anti-globalisation populist parties to seek compensation for the deprivation that they perceive modernisation has inflicted on them [4,8,31, for a discussion, see 32], so that, empirically, markers of lower relative objective status, such as low income, low education, blue collar work, and unemployment predict support for far-right parties [5,12,14,33]. Importantly, this research argues that relative deprivation constitutes a distinct causal pathway towards AfD support, summarized by the idea that ‘economic deprivation provides the motivational basis for right-wing radicalisation’ [9,34].

Part of the literature even argues that the direct link between anti-immigration attitudes and far-right support is driven by deprivation [10,33,35–37]. The argument is that feeling deprived, e.g., due to a low education, makes one vulnerable to competition from abroad, and this is what fosters anti-immigration attitudes in the first place [22,35,38]. In its simplest form, these arguments suggest that ‘far-right-wing ideologies act as psychological medicine against experiences of deprivation that are painful or anticipated with concern’ [39].

What these arguments have in common is that they insinuate that if one could somehow remove deprivation, AfD support would fall. Ultimately, this suggests deprivation not only explains AfD support directly, but also stands behind the direct link between anti-immigration attitude and AfD support [8,9,37,40]. From this, we derive our second hypothesis:

**Hypothesis 2**: *Variables that measure deprivation confound or moderate the effect between concern about immigration and AfD support*.

A popular variation of this argument is that ‘fear of social relegation’, rather than actual deprivation, explains AfD support [7]. Indeed, studies found that anxiety about one’s status is related to AfD support [41,42]. The causal argument why fear of relegation, rather than feelings of actual deprivation, could stand behind AfD support, is that individuals who feel socio-economically insecure are concerned that immigrants could receive the support that they may need for themselves due to their insecure position. In contrast, those who feel secure consider themselves sheltered from negative immigration effects that they might perceive. Some therefore argue that status anxiety, rather than actual deprivation, confounds or moderates the link between concern about immigration and AfD support [43,44]. This leads to our third hypothesis:

**Hypothesis 3**: *Variables that measure fear of deprivation confound or moderate the effect between concern about immigration and AfD support.*

In this case, either the direct relationship between concern about immigration and AfD support weakens after adjusting for fear of deprivation or there should be an interaction between fear of deprivation and concern about immigration, in the sense that those who feel deprived are particularly prone to support the AfD when they are concerned about immigration, while those who feel undeprived are not driven to the AfD even if they do fear immigration. Yet this hypothesis is contested as well, as some argue that a direct link between immigration concern and AfD support exists irrespective of such confounding or moderating [24]. By testing the three hypotheses above, we therefore aim to improve the extant literature by going beyond existing attempts to explain AfD support in several ways.

First, we test for the first time to which degree deprivation is even needed as an additional explanation and to which degree fear of immigration suffices to explain AfD support. Second, we do so by using random (between-individual) and fixed (within-individual) effects regressions. This enables us to draw more causal inferences, which not only show who supports the AfD, but also whether changes in the life of the same individual, such as becoming more concerned about immigration or feeling more deprived than before, lead to increasing AfD support. Third, we model how AfD support has changed across the years and thus whether the reasons that got individuals to support the AfD have changed. Fourth, we test whether concern about immigration works as an individual-level attitude or a population-level salience effect. Fifth, we measure both professed AfD support and actual voting. Sixth and last, we use an atheoretical data-mining approach that employs 257 additional variables in addition to our theory-driven variable selection, to make sure that we do not miss any relevant predictor of AfD support, to test whether anything explains AfD support in addition to or instead of what concern about immigration explains alone. Our results suggest however, that concern about immigration alone is such a strong predictor of AfD support, that comparatively little additional variation is explained by adding variables that measure deprivation. In addition, the direct relationship between concern about immigration and AfD support seems neither strongly confounded nor moderated by variables that measure deprivation.

## 2. Method and data

We first use logistic multivariate regressions, which explain AfD support yearly since the party exists and thus for each year including 2014–2024. This shows how the determinants of AfD support have changed annually. We then calculate logistic random effects regressions that explain AfD support only through concern about immigration. Subsequent models then show whether more total variation in AfD support is explained by adding deprivation-based variables, rather than only using concern about immigration, and how much the direct link between concern about immigration and AfD support weakens when adjusting for deprivation. This approach shows, respectively, whether deprivation explains more than immigration-concerns explains alone and whether deprivation stands behind, i.e., confounds, the link from immigration-concern to AfD support. We also use interaction effects to test moderation, i.e., to see whether those who feel deprived are more prone to support the AfD when they are also concerned about immigration. We then do the same with logistic fixed effects regressions, to additionally show how changes within the life of a person over time increase or decrease this person’s AfD support, to come to a more causal interpretation of whether concern about immigration is sufficient to explain why individuals support the AfD.

We standardised all independent variables to a standard deviation of 1, making their effects comparable. Since our dependent variable is binary, we use odds ratios to show effect sizes in regression tables. Odds ratios can approach infinity for positive effects, but are bounded at 0 for negative effects, which makes positive and negative effects difficult to compare. We therefore illustrate all effects using average marginal effects plots, which show by how many percentage points an individual’s probability to support the AfD changes if an independent variable increases by one standard deviation.

We use 11 waves of the German Socio-Economic Panel (SOEP), sampled between 2014 (the AfD’s founding year) and 2024 (the most recent data collection). This gives us 110,194 observations from 31,985 individuals (see Table OA1 in S1 File for all descriptives). Our dependent variable of AfD support is operationalised through responses to the question ‘Which party do you lean toward?’ (‘Welcher Partei neigen Sie zu?’). Responses take the value of 1 for those who support the AfD and 0 for those who mentioned any other party. In robustness tests, we also measure AfD support versus all other responses (rather than versus support for another party) and as actually having voted for the AfD in the last election.

We measure concern about immigration through responses to the question ‘How concerned are you about immigration to Germany’, which previous studies have argued to be a suitable measure of anti-immigration attitudes [22]. Answers range on a 3-point scale from ‘very concerned’, over ‘somewhat concerned’ to ‘not concerned at all’. We coded these as ordinal responses, with higher numbers reflecting increased concern about immigration. For a robustness test, we also measure salience of immigration concerns as the average response to this question on the level of state-years.

To operationalize deprivation, we calculated each respondent’s annual household net equivalent income percentile, so that, e.g., a percentile of 57 indicates that the respondent has more income than 57 per cent of all other respondents in this year. This variable’s positional nature fits the literature’s focus on relative deprivation, which makes sense in a developed country, where status is judged relative to others, including, crucially, immigrants [45]. We also measure status deprivation through education, as coded in the ISCED-1997-Classification. Excluding ‘in school’ leaves the categories ‘inadequately’, ‘general elementary’, ‘middle vocational’, ‘vocational + high school, ‘higher vocational’, ‘higher education’. Income percentile and education measure objective deprivation; subjective deprivation is measured through responses to three questions: ‘How satisfied are you with…’, ‘your household income?’, ‘your personal income?’, ‘your life, all things considered?’ Responses range from 0, ‘completely unsatisfied’, to 10, ‘completely satisfied’. Fear of deprivation is measured through answers to the question ‘How concerned are you about…’, ‘the economy in general’, and ‘your own economic situation?’ Responses vary from ‘not concerned at all’, over ‘somewhat concerned’ to ‘very concerned’. Note that with these variables, we only measure concern about immigration with a single variable, while we measure deprivation in a number of ways, to see if any type of deprivation might either explain AfD support in addition to or instead of concern about immigration.

Deriving these variables from the theories and ensuing hypotheses above runs the risk of overlooking a variable that is important, but has not yet been sufficiently conceptualized by the literature. In addition to the above-mentioned theoretically derived variables, we therefore also use a data mining approach as a robustness test, which uses a much broader and atheoretical 257 additional variables, to test if anything stands behind the direct link between immigration-concern and AfD support. Table OA1 in S1 File provides descriptives for the variables used in our main calculation.

## 3. Results

### 3.1. Factors explaining AfD support over time

**Fig 1:**
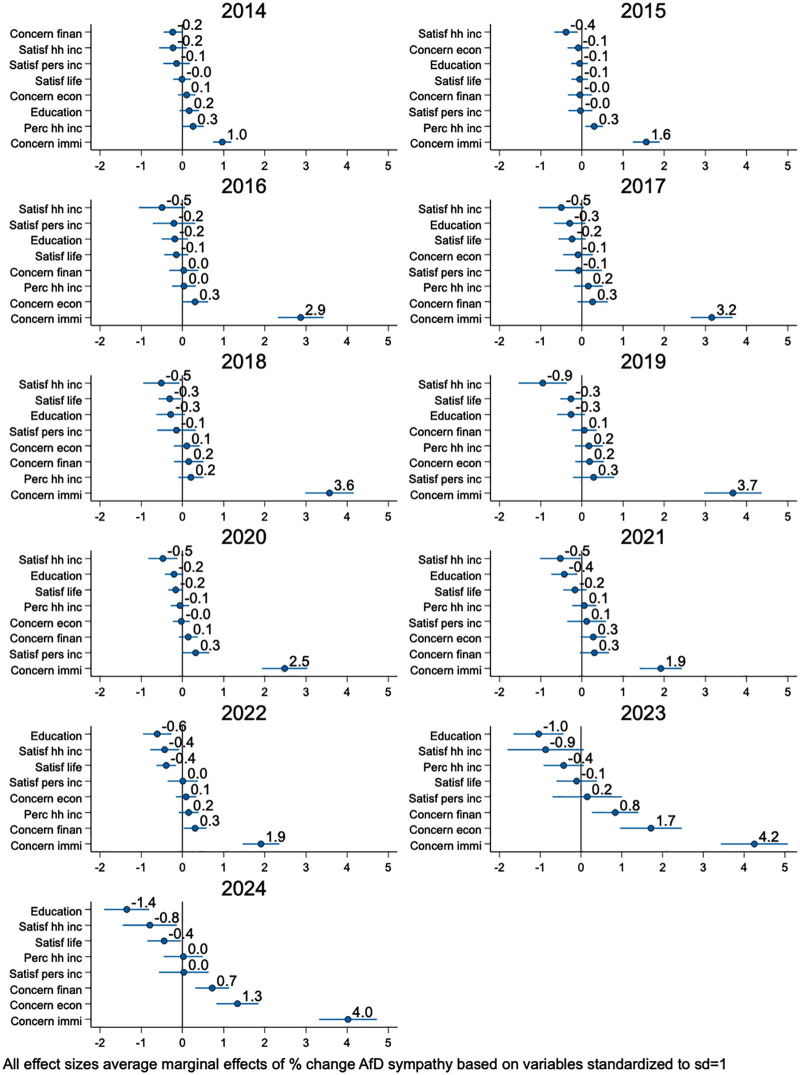
Average marginal effects of percentage likelihood to support AfD within each year.

Table OA2 in S1 File calculates logistic multivariate regressions that explain AfD support for every year since the AfD exists, using the variables mentioned above. Fig 1 shows effect sizes from these models, illlustrating how substantively and significantly each variable explains AfD support in each year.

Fig 1 shows how, in every single year since the AfD’s existence, concern about immigration is the most substantive predictor of AfD support. No other indicator predicts AfD support as significantly and consistently as immigration-concern does in every single year.

Directly after the AfD was founded, those who were one standard deviation more concerned about immigration had a 1 percentage point higher probability of supporting the AfD, holding all other variables constant. Model 1 in Table OA2 in S1 File shows that this amounts to an odds ratio of 2.94, suggesting that whose concern about immigration was one standard deviation higher in 2014 had an almost threefold chance to support the AfD. What is striking is that this effect of immigration-concern is more than three times stronger than any indicator of deprivation, even in the very first year of the AfD’s existence.

Over the 2010s, the effect of immigration-concern on AfD support only increased. Fig 1 indicates that whose concern about immigration was one standard deviation higher in 2019 had a 3.7 percentage points higher probability to support the AfD. Model 6 in Table OA2 shows that this amounts to 6.6-fold higher odds to support the AfD due to immigration-concern. Note also how no other predictor even reaches significance in explaining AfD support during this time, except satisfaction with household income, which seems to work against AfD support in some years. As Fig 1 shows, the link between immigration concern and AfD support then became temporarily weaker during the Covid-19 crisis. This changed again in 2023 and 2024, where one standard deviation concern about immigration led to a 3.8 percentage points higher probability to support the AfD, the strongest substantive effect of all years. This not only means that concern about immigration is consistently the strongest predictor of AfD support, but also that this seems to be increasingly the case.

While immigration-concern explains AfD support in every year, no other indicator is significant in every year. The second-strongest effect in the most recent year is that each standard-deviation increase in a respondent’s education decreases the probability to support the AfD by 1.4 percentage point in 2024, as the deprivation thesis would suggest. Yet in every year before 2021, the effect of education on AfD support is not even significant. The third-strongest effect in 2024 is that the probability to support the AfD increases by 1.3 percentage points for those who are more concerned about the economy. Yet this effect is not even significant in 8 out of 11 years.

That concern about immigration is the most substantive and significant explanation of AfD support during each year net of other variables is suggestive evidence in favour of hypothesis 1a and 1b. Yet it cannot show precisely how much additional variation in AfD support is explained through deprivation in addition to immigration-concern and how much the direct relationship between concern about immigration and AfD support is confounded by variables that measure deprivation. The following section shows this.

### 3.2. Random effects: Who supports the AfD?

We now turn to explaining AfD support across all years. The first model of Table 1 shows that who is one standard deviation more concerned about immigration has a more than ten-fold chance to support the AfD across all years. The model’s r^2^ shows that this concern about immigration alone explains about 8.9 per cent of all variation in AfD support.

**Table 1:** Random effects to explain who supports the AfD.

	(1)	(2)	(3)	(4)	(5)	(6)
	Concern immigration	Objective deprivation	Feared deprivation	All	Lagged deprivation	Interactions
AfD support						
Concern immi	10.66^***^	10.44^***^	10.43^***^	9.127^***^	9.064^***^	8.89^***^
	(38.73)	(35.15)	(36.13)	(34.35)	(21.97)	(32.25)
Education		.4633^***^		.4655^***^	.5207^***^	.3945^***^
		(−13.58)		(−13.57)	(−5.96)	(−10.27)
Perc hh inc		.778^***^		.7866^***^	.7957^**^	.6536^***^
		(−5.52)		(−5.24)	(−2.98)	(−5.64)
Satisf hh inc		.6621^***^		.6869^***^	.7155^***^	.7233^***^
		(−7.54)		(−6.76)	(−3.50)	(−3.69)
Satisf pers inc		1.014		1.033	1.113	.9999
		(0.24)		(0.58)	(1.14)	(−0.00)
Satisf life		.8026^***^		.844^***^	.843^**^	.8415^**^
		(−6.46)		(−4.92)	(−2.97)	(−3.03)
Concern finan			1.401^***^	1.104^*^	1.27^***^	1.006
			(9.06)	(2.51)	(3.70)	(0.08)
Concern econ			1.534^***^	1.58^***^	1.454^***^	1.47^***^
			(11.42)	(11.97)	(6.40)	(5.88)
Education * Concern immi						1.314^***^
						(3.73)
Perc hh inc * Concern immi						1.264^***^
						(3.78)
Satisf hh inc * Concern immi						.9503
						(−0.68)
Satisf pers inc * Concern immi						1.036
						(0.46)
Satisf life * Concern immi						1.009
						(0.19)
Concern finan * Concern immi						1.106
						(1.68)
Concern econ * Concern immi						1.07
						(1.28)
						
lnsig2u	21.46^***^	21.62^***^	21.38^***^	21.38^***^	30.12^***^	22.68^***^
	(73.66)	(73.10)	(70.85)	(72.81)	(50.43)	(64.91)
N	110194	110194	110194	110194	64980	110194
N individuals	31985	31985	31985	31985	17468	31985
ll	−12039	−11759	−11896	−11645	−5324	−11585
aic	24083	23535	23802	23310	10667	23205
bic	24112	23612	23850	23406	10758	23368
r^2^	.08895	.1101	.09974	.1187	.5971	.1232

All effect sizes standardized to 1 = 1 sd; Table shows odds ratios based on Stata xtlogit re procedure with robust standard errors.

* *p* < 0.05, ** *p* < 0.01, *** *p* < 0.001.

Model 2 adds the five variables that measure deprivation. Model 2 explains 11 percent of variation in AfD support, which is only 24 per cent higher than what Model 1 explained through concern about immigration alone. This supports Hypothesis 1a, that compared to explaining AfD support with concern about immigration alone, adding variables that measure deprivation explains little additional variation.

In addition, someone who is more concerned about immigration, but not particularly deprived, still has a 10-times higher chance to support the AfD. Thus, the direct relationship between concern about immigration and AfD support is not strongly confounded by variables that measure deprivation, validating Hypothesis 1b that the magnitude and direction of the relationship between AfD support and concern about immigration does not change substantially, irrespective of variables that measure deprivation. In turn, this disconfirms Hypothesis 2, as variables that measure deprivation do not strongly confound or moderate the effect between concern about immigration and AfD support.

Simply put, this means that judged against the baseline of explaining AfD support through immigration-concern alone, explaining AfD support through deprivation is neither a strong alternative, nor a strong supplemental explanation.

Model 3 shows that someone who is more concerned about immigration, but not particularly fearful of deprivation, still has 10-times higher chance to support the AfD. Adding variables that measure feared deprivation does not explain much more variation in AfD support than concern about immigration explains alone, as Model 3’s r^2^ is only 12 percent higher than Model 1’s. This again confirms Hypothesis 1a. Also, that the direct link between immigration concern and AfD support remains intact validates Hypothesis 1b and invalidates Hypothesis 3. Neither actual nor feared deprivation, it seems, explain much more variation in AfD support than immigration-concern alone, and thus explaining AfD support through either felt or feared deprivation is neither a strong alternative, nor a strong supplemental explanation to explaining AfD support through concern about immigration alone.

**Fig 2:**
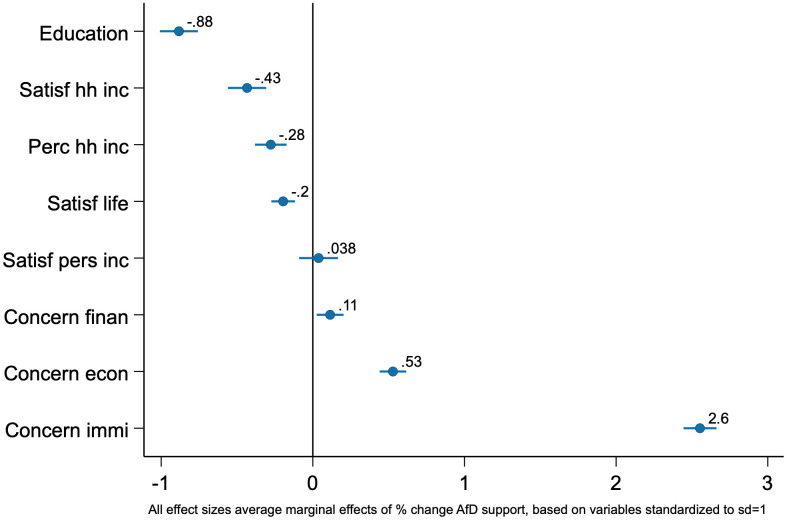
Model 4 standardized effects of all variables on AfD support.

Model 4 takes up all variables simultaneously. Keep in mind that Table 1 shows odds ratios which can approach infinity for positive effects, but are bounded at 0 for negative effects, which makes positive and negative effects difficult to compare. Fig 2 therefore visualizes the effects of Model 4 by converting odds ratios into average marginal effects, showing how one standard deviation increase of each independent variable is related to the probability of AfD support, irrespective of changes in the other variables. While the tables thus show effects as odds ratios (whose lower bound is 0 and upper bound is infinity), Fig 2 converts this into average marginal effects, which shows effects measured as changes in the probability to support the AfD on a scale from 0–1. Model 4 in Table 1 shows that, even adding both actual and feared deprivation only increases the total explained variation of AfD support by 33 percent compared to explaining AfD support merely through concern about immigration, supporting Hypothesis 1a. In addition, the direct link from concern about immigration to AfD support remains intact, even when adjusting for objective and feared deprivation, supporting Hypothesis 1b and invalidating Hypothesis 2 and 3.

The overpowering influence of concern about immigration on AfD is also illustrated by Fig 2, which shows that being one standard deviation more concerned about immigration translates into 2.4 percentage points more support for the AfD, an effect that is three times stronger than the second-strongest effect (of having a higher education).

Models 5 and 6 test alternative specifications to verify the main results. First, Model 5 uses measures of lagged deprivation, defined as the deprivation that an individual has experienced last year. It does this because it is possible that last year’s deprivation is somehow an antecedent to this year’s fear of immigration and AfD support. Yet, as can be seen both from its explained variation (which is only 11 percent) such a setup does not explain more than the previous model (supporting Hypothesis 1a), and the direct link between concern about immigration and AfD support is also not diminished after accounting for prior deprivation (supporting Hypothesis 1b). At the same time, this shows that even temporarily prior deprivation does not seem to confound the link between concern about immigration and AfD support (invalidating Hypothesis 2 and 3).

Next, the last Model 6 interacts each deprivation variable with concern for immigration to test the moderation part of each hypothesis. Yet comparing Model 6 to the main Model 4 shows that it does not explain much more variation in AfD support (supporting Hypothesis 1a) and does not strongly diminish the direct link from concern about immigration to AfD support (supporting Hypothesis 1b). Further, what is striking is that the interaction effects go in the opposite direction of what a losers of modernization thesis would predict, as it is the highly educated and those who perceive themselves to have a high income who support the AfD more when they fear immigration. Thus, it is not the deprived who are driven to the AfD through a fear of immigration, but, quite to the contrary, the higher-educated and those who feel well-off. All regressions therefore seem to suggest the same message: It is concern about immigration, rather than feeling deprived, that explains AfD support. Conversely, feeling deprived is neither a good additional nor alternative explanation for explaining AfD support, compared to explaining it concern about immigration alone.

**Fig 3:**
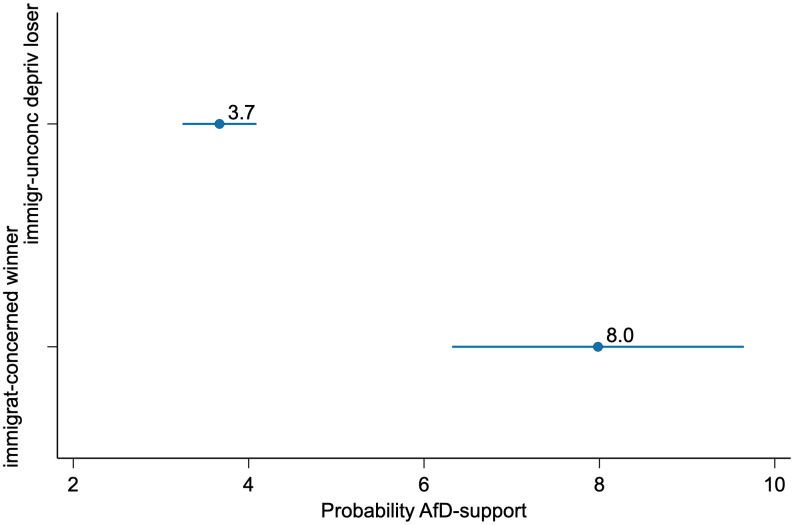
Predicted probabilities of AfD support for immigration-concerned modernisation winner versus immigration-unconcerned deprived modernisation loser.

To illustrate this, Fig 3 shows predicted probabilities of two statistically constructed individuals. The upper line of Fig 3 shows the predicted probability to support the AfD for 1) an “immigration-unconcerned deprived loser of modernization”, who is maximally deprived (lowest education, poorest income percentile, lowest satisfaction with personal income, household income, life overall and maximally concerned about own finances and Germany’s economy), who thus should support the AfD, yet feels “not concerned at all” about immigration and thus should not support the AfD. Indeed, this maximally deprived but immigration-unconcerned individual only has a probability of 3.7 percent to support the AfD.

We contrast this to the probability of AfD support of 2) an “immigration-concerned non-deprived winner of modernization”, who is the opposite of our first individual in every way, being “very concerned” about immigration, yet also being in the highest income percentile, having the highest education, highest possible satisfaction with household income, personal income and life overall, as well as being entirely unworried about personal finances or the overall economy. From the “losers of modernization” thesis, this person should not support the AfD. Yet this person’s probability to support the AfD is 8 percent and therefore more than twice as high as that of our statistically constructed “loser of modernization”, who is deprived in every way, yet unconcerned about immigration.

In this way, someone who is concerned about immigration while being totally privileged still has more than twice the probability to support the AfD as someone who is not concerned about immigration, even though he is totally deprived. In this sense, whoever is concerned about immigration supports the AfD significantly and substantively more, regardless of deprivation, while someone who is unconcerned about immigration supports the AfD significantly less, however deprived this person may feel.

### 3.3. Fixed effects: Which changes explain that the same person supports the AfD more than before?

While the preceding regressions have shown who supports the AfD compared to someone who does not, Table 2 shows the results of fixed effect regressions, which indicate under which conditions someone who has not supported the AfD so far starts to do so and thus which changes in the life of an individual are related to whether or not this individual starts to support the AfD. Since these regressions model intra-individual change, they hold all inter-individual differences constant, allowing for a more causal inference of AfD support. The disadvantage of this is that it can only draw variation from those 936 individuals who changed their AfD support between 2014 and 2024, resulting in merely 3597 observations, and thus less than 5 per cent of the cases that the random effects models above could analyse. The fixed effect specification also means that it makes no sense to include education as a predictor. Because education in a fixed effects model does not show differences in education between differently educated individuals, but the effect of individually increasing education, which conflates life cycle and actual education effects.

**Table 2:** Fixed effects to explain which changes in life increase AfD support.

	(1)	(2)	(3)	(4)	(5)	(6)
	Concern immigration	Objective deprivation	Feared deprivation	All	Lagged deprivation	Interactions
AfD support						
Concern immi	2.71^***^	2.69^***^	2.63^***^	2.61^***^	2.32^***^	2.66^***^
	(12.33)	(11.03)	(13.56)	(12.12)	(5.94)	(14.24)
Perc hh inc		1.08		1.09	1.01	1.05
		(0.90)		(0.93)	(0.05)	(0.39)
Satisf hh inc		.891		.89	1.01	.93
		(−1.43)		(−1.26)	(0.06)	(−0.66)
Satisf pers inc		1.06		1.05	1.03	.931
		(0.62)		(0.51)	(0.26)	(−0.58)
Satisf life		.944		.962	.878^*^	1
		(−1.24)		(−0.71)	(−2.09)	(0.02)
Concern finan			.946	.932	1.04	.855
			(−1.04)	(−1.08)	(0.47)	(−1.29)
Concern econ			1.37^***^	1.37^***^	1.21^**^	1.4^***^
			(7.79)	(7.03)	(2.59)	(4.30)
Perc hh inc * Concern immi						1.04
						(0.58)
Satisf hh inc * Concern immi						.953
						(−0.56)
Satisf pers inc * Concern immi						1.14
						(1.55)
Satisf life * Concern immi						.962
						(−0.73)
Concern finan * Concern immi						1.1
						(1.03)
Concern econ * Concern immi						.975
						(−0.40)
N	4519	4519	4519	4519	1798	4519
N individuals	936	936	936	936	376	936
ll	−1561	−1559	−1533	−1531	−614	−1529
aic	3125	3127	3073	3076	1241	3083
bic	3131	3159	3092	3121	1280	3167
r^2^	.0848	.0864	.101	.103	.0669	.104

All effect sizes standardized to 1 = 1 sd, Table shows odds ratios based on Stata xtlogit fe procedure with standard errors based on bootstrapping, as xtlogit fe does not allow robust standard errors.

* *p* < 0.05, ** *p* < 0.01, *** *p* < 0.001.

Except for exluding education, the models of Table 2 are set up precisely as in Table 1. Substantively, Model 1 of Table 2 shows that when the same person is one standard deviation more concerned about immigration, then this same person’s chance to support the AfD almost triples. This alone explains 8.5 percent of all variation of whether the same individual support the AfD.

Model 2 adds measures of deprivation. Yet, this only increases the total explained variation of AfD support by a mere 2 percent, supporting Hypothesis 1a. Also, the direct link between immigration concern and AfD support remains virtually the same after accounting for deprivation, supporting Hypothesis 1b. It is unsurprising that individually changing deprivation does not explain individually changing AfD support, as not a single measure of individual change in deprivation is related to individual change in AfD support, invalidating Hypothesis 2.

Model 3 adds measures of feared deprivation. Compared to the base model, this only increases total variation by 19 percent. Also, the direct link between individually changing immigration concern and individually changing AfD support remains intact. Again, this tends to support Hypothesis 1a and 1b, while invalidating Hypothesis 3.

**Fig 4:**
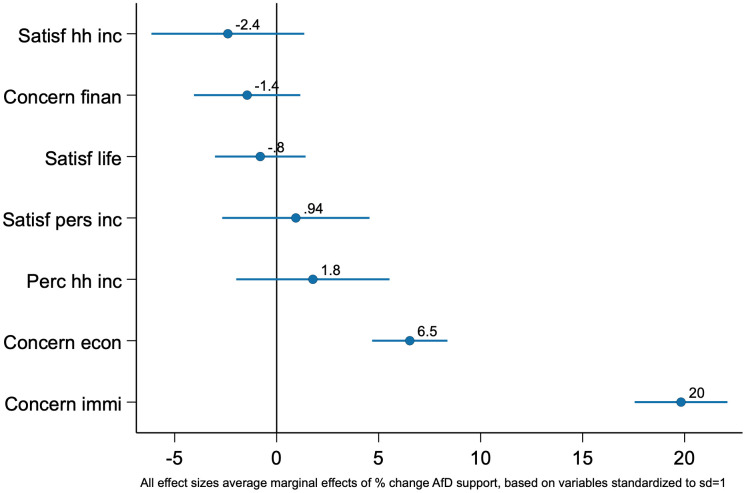
Effect sizes of Model 4 on which changes in life increase AfD support (fixed effects).

Model 4 adds all variables. First, it shows that all deprivation variables combined only explain 21 percent more variation in individually changing AfD support than individually changing concern about immigration explains alone. Second, even adding all deprivation variables simultaneously leaves the direct relationship between individually changing concern about immigration and individually changing AfD support intact. Again, this is strong support for hypotheses 1a and 1b, while contradicting hypothesis 2 and 3. To illustrate how much stronger individual changes in immigration concern explain individually changing AfD support compared to all other variables, Fig 4 shows that if the same individual becomes one standard deviation more concerned about immigration, her probability to support the AfD increases by 20 percentage points. The only other significant effect is that if the same individual becomes one standard deviation more concerned about the economy, her probability to support the AfD changes by 6.5 percentage points. All other changes in the life of the same person do not even decrease or increase her probability to support the AfD significantly.

Note that concern about the economy, the strongest deprivation variable, does not even measure deprivation directly, as one can be concerned about the economy, while individually being in a financially comfortable situation. Thus, it is striking how all variables that measure deprivation directly, are not even significantly related to support for the AfD. In this sense, one can say that if the same individual feels more deprived than before, her probability to support the AfD does not increase. Conversely, if the same individual feels less deprived than before, she – on average – does not support the AfD less. Conversely, changes in an individual’s concern about immigration translate into large changes in AfD support.

Models 5 and 6 again test alternative specifications to verify the main results. Model 5 measures whether having experienced increased deprivation last year turns the same individual towards the AfD this year. Yet, as can be seen both from its explained variation (which is only 6.7 percent), this does not explain more than the previous model (supporting Hypothesis 1a), and the direct link between concern about immigration and AfD support is also not diminished after accounting for prior deprivation (supporting Hypothesis 1b). At the same time, it shows that even temporarily prior changing deprivation does not seem to confound the link between concern about immigration and consequent AfD support (invalidating Hypothesis 2 and 3).

Next, the last Model 6 interacts each deprivation variable with concern for immigration to test the moderation part of each hypothesis. Yet comparing it to the main model 4 shows that it does not explain much more variation in AfD support (supporting Hypothesis 1a) and does not strongly diminish the direct link from concern about immigration to AfD support (supporting Hypothesis 1b). Every single interaction effect is insignificant, meaning that the same individual does not support the AfD more when experiencing increased concern about immigration while being, e.g., decreasingly satisfied with one’s income.

Regressions that analyse change over time within the life of the same individual therefore support the message from Table 1, which also compared individuals rather than only within-individual change: It is concern about immigration, rather than feeling deprived, which explains AfD support. Conversely, feeling deprived is neither a good additional nor alternative explanation compared to explaining AfD support through concern about immigration alone. As we have seen, this is both true when focussing on changes in the life of a person, as Table 2 has done, as well as when comparing individuals, as Table 1 has done.

### 3.4. Robustness tests

One could question our results by arguing that immigration-concern is not merely an individual-level variable, but a societal phenomenon. An individual might not be worried about immigration herself, but perceive all others to be, and thus sympathize with the AfD due to perceiving a widespread concern about immigration, while not being concerned herself. From this perspective, it is not individual immigration-concern that explains AfD support, but the topic’s broader salience. To test this, we included the annual regional average immigration concern on the level of German regions (Bundesländer) and years. We then used logistic multilevel regressions that nest individuals non-hierarchically in states and years. Results can be found in Table OA3 in S1 File. The results show that both individual concern about immigration, as well as annual state-level average concern about immigration promote AfD support. Thus, not only individual concern about immigration influences support for the AfD. Instead, the broader concern about immigration in an individual’s context has an additional independent effect. The latter context effect is relatively weak compared to the main effect of individual concern and also barely significant, also because it is an annual state-level variable, which means it has relatively few independent cases. What may be more important in the context of our research question, however, is that adding variables that measure deprivation again does not strongly increase the explanatory effect of individual or context-level concern about immigration. Notably, Table OA 3 Model 1, which only contains individual- and context-level immigration-concern, already explains 86 percent of what Model 2 explains, which includes the seven additional variables that measure deprivation. In addition, the direct link from both individual- and context-level immigration-concern to AfD support does not strongly diminish after including deprivation-related variables. Indeed, in the case of population-level concern about immigration, the relationship becomes slightly stronger. This shows that, if anything, our main results are rather conservative; and it supports our main result that deprivation variables neither explain much more variation in AfD support than immigration-concern explains alone, nor does deprivation seem to stand behind the direct link from concern about immigration to AfD support. Simply put, deprivation variables are therefore neither a strong additional nor alternative explanation to explaining AfD-support through immigration-concern alone, and this becomes even more obvious when conceptualizing immigration-concern as a context variable, rather than only as an individual-level variable.

Second, it could make sense to code AfD support more broadly. In our main calculations, we have compared individuals who support the AfD rather than any other political party. For the results of Table OA4 in the S1 File, we have coded who supports the AfD against all other responses, including not supporting any political party at all. If we code AfD support in this way, immigration-concern explains 85 percent of the variation that a model including nimmigration-concern plus deprivation explains (compare the change of r^2^ from Model 1 to Model 2 in Table OA4). And, as in our main calculations, the direct relationship between immigration-concern and AfD support hardly changes after including deprivation variables. For Models 3 and 4 in Table OA4, we have additionally coded AfD support through having voted for the AfD in the last election. For this alternative dependent variable, immigration-concern alone explains slightly more than 90 percent of the variation that immigration-concern and deprivation explain together (see the difference between r^2^ from Model 3 and Model 4 in Table OA4) and again, the direct relationship between immigration-concern and having voted for the AfD hardly diminishes after including deprivation-variables.

Third, we have adjusted for the demographic variables that are typically mentioned in the literature to explain AfD support, such as being a man [7,17], being older/younger [12,20], or living in a specific German states [6,8]. We therefore use gender, age and age squared, as well as residency in each of Germany’s 16 federal states as controls (see Table OA5 in S1 File). This adjustment changes nothing with respect to our main results: immigration-concern still explains 80 percent of what immigration-concern and deprivation explain together and the direct link between immigration-concern and AfD support hardly diminishes after including deprivation variables. Since all robustness tests thus showed even stronger support for Hypothesis 1a and 1b, and against Hypothesis 2 and 3, than our main calculations did, our main results should be considered a lower bound on how strongly immigration-concern alone influences AfD support.

Fourth, in addition to our theoretical selection of variables based on the literature above, we have complemented our main analysis through a purposefully atheoretical data mining approach. For this, we estimated whether 257 additional variables, basically any attitudinal variable that could conceivably have any explanatory value, explain AfD support in addition to what immigration concern explains alone. While this atheoretical approach would make no sense if used alone, it can be helpful to complement the theory-based selection of variables above, in case the literature has overlooked an important deprivation variable that explains AfD support in addition to concern about immigration. Yet Table OA5 in the S1 File shows that those variables that explain most variation in AfD support in addition to concern about immigration are mostly (but not entirely) themselves related to immigration-concerns, while hardly ever being deprivation-related. For example, the strongest additional influence on AfD support next to immigration-concern are (in that order): 1) agreeing that refugees make Germany a worse place to live, 2) being unvaccinated against Covid-19, 3) agreeing that the influence of refugees on cultural life is negative, 4) feeling unconnected to Europe, 5) agreeing that refugees are negative for the economy, 6) agreeing that refugees are a risk rather than opportunity, which is followed by anti-LGBTQ attitudes, refusal to consume “established” media and feeling discriminated due to one’s political attitude. These are, broadly speaking, cultural variables, in the sense that they reflect views on the world, more than socio-economic deprivation. The first variable that could be conceivably understood as such comes in 15th place, and it is ‘worries that one cannot keep up with technological progress’. Thus, even if we expand our universe of attitudes to explain AfD support without any theoretical basis, purely based on all available data, then our main conclusion still holds that the explanatory strength of concern about immigration is hardly confounded by any form of deprivation, remaining the strongest predictor for AfD-support. Indeed, this robustness test suggests that immigration concern is not only more important than socio-economic deprivation to explain AfD-support, but that cultural attitudes writ large are generally more important than socio-economic conditions to explain AfD support.

Fifth, an argument could be made that whoever is associated with the category “worker” supports the AfD [cf. 46,47]. We therefore coded who is a worker using the SOEP’s occupational scheme (see last heading “Are workers closer to the AfD?” in S1 File). Yet knowing whether someone is a worker only increases the total explained AfD support by 3 percent, compared to knowing whether someone is concerned about immigration. Similar to our main results, knowing whether someone could be “deprived”, in this case by being a worker, helps little to understand AfD support beyond what concern about immigration explains alone, and does not explain why immigration-concern leads to AfD-support. Additionally, we restricted our analysis to workers, to test whether these are particularly drawn to the AfD when they are concerned about immigration. However, a worker’s chance to support the AfD increased about 8-fold when being one standard deviation more concerned about immigration, which is almost the same as our main effect. Therefore, belonging to the group of “workers” has no specific effect on the relationship between AfD support and concern about immigration.

## 4. Discussion

Our findings indicate that deprivation explains little additional variation compared to explaining AfD support through immigration concern alone (supporting Hypothesis 1a). The highly significant direct link between immigration concern and AfD support also hardly changes after accounting for deprivation (supporting Hypothesis 1b). Temporally preceding deprivation variables show that the direct link between fear of immigration and AfD support does not appear to be due to prior deprivation. Interaction effects show that those who feel more deprived are not particularly driven to the AfD when concerned about immigration. Individual fixed effects show that the same person does support the AfD more when being more concerned about immigration, but whether she additionally experiences increased deprivation explains little. That neither perceived nor feared deprivation strongly confound nor moderate effects of immigration-concern on AfD-support invalidates Hypothesis 2 and 3.

Our results also showed how an individual who is non-deprived in every way, yet is concerned about immigration, is still about twice as likely to support the AfD compared to someone who is deprived in every way, but unconcerned about immigration. The prediction model “tell me your concerns about immigration and I know whether you support the AfD, irrespective of your socio-economic condition” therefore works well, while the prediction model “tell me about your socio-economic condition and I know whether you support the AfD, irrespective of your immigration-concern” does not work well. This can be summarized as the statement that deprivation variables neither significantly complement nor replace the direct link between immigration concern and AfD support.

More than previous studies did, this questions the “losers of modernisation” hypothesis [7,12,18,24–26], as it undermines claims that anti-immigration attitudes are insufficient to explain AfD support. In fact, our results suggest the opposite: empirical studies that use socio-economic variables without anti-immigration attitudes might be fundamentally misguided, as effects from such studies may only appear because socio-economic variables are confounded with immigration-concern, rather than the other way around [4,8,9,33,35,45]. Simply put, our results raise the question whether actual or feared deprivation play any significant role in explaining far-right party support.

While our results are quite clear in this regard, they lack a causal explanation. Notably, they raise the question why a strong direct link between concern about immigration and AfD support is irreducible to deprivation. Many attempts to explain such a direct connection merely shift the burden of explanation. For instance, it is widely claimed that AfD supporters are less satisfied with democracy [19,20,33,42,48–50]. Yet this merely shifts the question to asking: Why are AfD supporters less satisfied with democracy? The question therefore is why concern about immigration could be a genuine cause of AfD-support that is irreducible to prior influences. A straightforward answer is that AfD supporters hold a “nativist” worldview, which sees countries as constituted of an ethnoculturally defined native group, whose homogeneity needs to be protected against non-native elements [32] that are understood as an external “Other” [11,18,51].

Why some hold this view can be explained by Moral Foundations Theory, which suggests that the morality of left-wing individuals is rooted in the two notions of care and fairness, which are individualizing moral foundations, as they emphasize the universality of individual rights. Subscribers to these moral foundations therefore argue that every individual has the same right to be cared for, and that it is unfair if an individual is born into a poor country, so that this person should have the right to immigrate into a richer country.

In contrast, supporters of the political right build their moral intuitions on loyalty, authority, and sanctity. These three moral foundations are believed to bind individuals into an ingroup, which adherents to these moral beliefs want to protect from what they perceive as an outgroup [27–30]. Consequently, individuals who subscribe to these moral foundations are more likely to be swayed by arguments that immigrants dilute the belief in ingroup authorities, endanger the community and introduce divergent notions of what is considered sacred [52]. It is then no surprise that individuals who subscribe to the binding moral foundations of loyalty, authority and sanctity support a party that promises to safeguard what they consider important [53].

Rather than assuming that status deprivation really stands behind AfD support, we therefore suggest that an alternative theory is more straightforward, which is that the AfD makes a political offer that resonates with the “binding” moral foundations of some. Consequently, future research could investigate whether, and to what degree, moral foundations of loyalty, authority, and sanctity explain anti-immigration attitudes and thereby AfD support.

Another obvious avenue for future research would be to explore whether concern about immigration is similarly sufficient to explain far-right party support in other countries. Germany might be a special case, as the AfD emerged as a party of economically liberal and relatively privileged university professors, yet has since shifted so far to the far-right [12] that even other European far-right parties refuse to collaborate with it. It therefore remains to be seen whether it is an exception that concern about immigration alone can explain the success of a right-wing party with little additional variation being due to deprivation. Yet by largely rejecting the losers of modernization hypothesis for Germany, we hope that we have advanced an interesting new hypothesis, namely that the direct cultural link between concern about immigration and AfD support is so strong that socio-economic deprivation is neither a good additional nor alternative explanation.

## Supporting information

S1 File Online Annex AfD with Stata code to reproduce everything. (DOCX)

S2 File Stata code to reproduce. (PDF)
