# Supplementary material for: Supporters of Germany’s far-right AfD party are concerned about immigration rather than feeling deprived
Source: PLoS One. 2026 Jul 21;21(7):e0350403. doi: 10.1371/journal.pone.0350403 (PMC13387537; doi:10.1371/journal.pone.0350403)
Supplement: S1 File — (DOCX) [file pone.0350403.s001.docx]

Online Annex

# Descriptives

Table OA 1: Descriptives

| Variable | N | Mean | SD | Min | Max |  |
| --- | --- | --- | --- | --- | --- | --- |
|  |  |  |  |  |  |  |
| AfD support | 110194 | 0.05 | 0.22 | 0.00 | 1.00 |  |
| Concern immi | 110194 | 1.91 | 0.76 | 1.00 | 3.00 |  |
| Satisf hh inc | 110194 | 7.32 | 2.07 | 0.00 | 10.00 |  |
| Satisf pers inc | 110194 | 6.91 | 2.33 | 0.00 | 10.00 |  |
| Satisf life | 110194 | 7.53 | 1.61 | 0.00 | 10.00 |  |
| Percentile hh net equiv inc | 110194 | 57.59 | 28.44 | 1.00 | 100.00 |  |
| Education PGISCED 97 scale | 110194 | 4.28 | 1.48 | 1.00 | 6.00 |  |
| Concern own finan | 110194 | 1.62 | 0.66 | 1.00 | 3.00 |  |
| Concern Ger economy | 110194 | 1.96 | 0.63 | 1.00 | 3.00 |  |

Table OA 2: Annual regressions

|  | (1) | (2) | (3) | (4) | (5) | (6) | (7) | (8) | (9) | (10) | (11) |
| --- | --- | --- | --- | --- | --- | --- | --- | --- | --- | --- | --- |
|  | 2014 | 2015 | 2016 | 2017 | 2018 | 2019 | 2020 | 2021 | 2022 | 2023 | 2024 |
| AfD support |  |  |  |  |  |  |  |  |  |  |  |
| Concern immi | 2.94^***^ | 4.76^***^ | 6.01^***^ | 4.97^***^ | 5.83^***^ | 6.58^***^ | 7.45^***^ | 4.54^***^ | 4.27^***^ | 4.26^***^ | 3.89^***^ |
|  | (9.14) | (10.06) | (7.90) | (11.37) | (14.38) | (14.39) | (15.22) | (9.81) | (10.44) | (10.52) | (11.80) |
| Satisf hh inc | .773 | .68^**^ | .734 | .775 | .776^*^ | .614^***^ | .682^**^ | .662^*^ | .72^**^ | .743 | .765^*^ |
|  | (-1.33) | (-2.81) | (-1.90) | (-1.78) | (-2.23) | (-3.55) | (-2.78) | (-2.12) | (-2.59) | (-1.87) | (-2.45) |
| Satisf pers inc | .853 | .967 | .879 | .959 | .931 | 1.16 | 1.3 | 1.1 | 1.01 | 1.05 | 1.01 |
|  | (-0.86) | (-0.23) | (-0.77) | (-0.28) | (-0.62) | (1.13) | (1.92) | (0.48) | (0.05) | (0.35) | (0.11) |
| Satisf life | .988 | .95 | .911 | .886 | .858^*^ | .872^*^ | .875 | .876 | .74^***^ | .964 | .86^*^ |
|  | (-0.10) | (-0.50) | (-1.03) | (-1.41) | (-2.35) | (-2.04) | (-1.81) | (-1.17) | (-4.27) | (-0.43) | (-2.17) |
| Perc hh inc | 1.33 | 1.35^**^ | 1.02 | 1.08 | 1.11 | 1.09 | .953 | 1.05 | 1.12 | .864 | 1.01 |
|  | (1.80) | (2.91) | (0.25) | (0.88) | (1.34) | (1.02) | (-0.53) | (0.40) | (1.33) | (-1.73) | (0.09) |
| Education | 1.2 | .947 | .891 | .86 | .869 | .874 | .848 | .714^**^ | .628^***^ | .701^***^ | .633^***^ |
|  | (1.42) | (-0.54) | (-1.26) | (-1.58) | (-1.68) | (-1.60) | (-1.87) | (-2.99) | (-5.11) | (-3.71) | (-5.69) |
| Concern finan | .768^*^ | .954 | 1.02 | 1.14 | 1.08 | 1.03 | 1.12 | 1.27 | 1.26^*^ | 1.33^**^ | 1.28^***^ |
|  | (-2.06) | (-0.32) | (0.18) | (1.41) | (0.84) | (0.38) | (1.23) | (1.79) | (2.44) | (3.00) | (3.51) |
| Concern econ | 1.12 | .918 | 1.21^*^ | .954 | 1.05 | 1.1 | .979 | 1.24 | 1.07 | 1.79^***^ | 1.57^***^ |
|  | (0.94) | (-0.64) | (2.05) | (-0.50) | (0.66) | (1.09) | (-0.25) | (1.87) | (0.68) | (5.00) | (5.30) |
| N | 10945 | 9277 | 8677 | 9672 | 9775 | 10523 | 10092 | 7251 | 9591 | 6921 | 8550 |
| r2_p | .104 | .167 | .203 | .192 | .259 | .291 | .312 | .299 | .304 | .344 | .291 |

All effect sizes standardized to 1=1 sd

^*^ *p* < 0.05, ^**^ *p* < 0.01, ^***^ *p* < 0.001

# Robustness tests

## State-level effects

Table OA 3: Immigration-concern as state-level effect

|  | (1) | (2) |
| --- | --- | --- |
|  | Concern_immigration | All |
| AfD support |  |  |
| Concern immi | 5.858^***^ | 4.843^***^ |
|  | (70.87) | (61.36) |
| Popul concern immi | 1.816^*^ | 1.884^**^ |
|  | (2.51) | (2.63) |
| Education |  | .7173^***^ |
|  |  | (-18.09) |
| Perc hh inc |  | .9769 |
|  |  | (-1.28) |
| Satisf hh inc |  | .7882^***^ |
|  |  | (-9.04) |
| Satisf pers inc |  | 1.012 |
|  |  | (0.46) |
| Satisf life |  | .8969^***^ |
|  |  | (-7.00) |
| Concern finan |  | 1.118^***^ |
|  |  | (6.09) |
| Concern econ |  | 1.136^***^ |
|  |  | (7.19) |
| / |  |  |
| var(_cons[_all>syear]) | 13.49^*^ | 16.21^*^ |
|  | (2.06) | (2.07) |
| var(_cons[state]) | 1.222^*^ | 1.23^*^ |
|  | (2.49) | (2.50) |
| N | 109415 | 109415 |
| N_clust |  |  |
| pr2 | .2191 | .2523 |

All effect sizes standardized to 1=1 sd

^*^ *p* < 0.05, ^**^ *p* < 0.01, ^***^ *p* < 0.001

## Different coding of AfD support

Our main regressions operationalized AfD-support through those who leaned towards the AfD rather than another political party. One could also compare AfD-supporters to those who support another political party or no party at all; second, one could measure revealed preferences through having voting for the AfD in the last election. Both operationalizations have disadvantages. Measuring AfD-support versus support for no party introduces the additional distinction of supporting any political party (including the AfD) versus supporting none. Our second alternative indicator, having voted for the AfD, is only available after federal elections and mixes a measure of support with strategic voting considerations. Yet, we conceptualize AfD-support in both ways, as detailed in Table OA4.

Table OA 4: Different coding of AfD support

|  | (1) | (2) | (3) | (4) |
| --- | --- | --- | --- | --- |
|  | afd  pref  vs  all  oth | afd  pref  vs  all  oth  depriv | afd  vote | afd  vote  depriv |
| main |  |  |  |  |
| Concern immi | 4.77^***^ | 4.17^***^ | 5.69^***^ | 4.93^***^ |
|  | (40.62) | (36.96) | (33.35) | (30.80) |
| Education |  | .779^***^ |  | .722^***^ |
|  |  | (-7.31) |  | (-7.68) |
| Perc hh inc |  | 1.03 |  | .853^***^ |
|  |  | (0.94) |  | (-4.05) |
| Satisf hh inc |  | .857^***^ |  | .739^***^ |
|  |  | (-4.35) |  | (-5.28) |
| Satisf pers inc |  | 1.03 |  | 1.1 |
|  |  | (0.77) |  | (1.75) |
| Satisf life |  | .891^***^ |  | .936 |
|  |  | (-4.95) |  | (-1.88) |
| Concern finan |  | .968 |  | .995 |
|  |  | (-1.31) |  | (-0.12) |
| Concern econ |  | 1.46^***^ |  | 1.13^***^ |
|  |  | (15.06) |  | (3.44) |
| / |  |  |  |  |
| lnsig2u | 8.1^***^ | 8.04^***^ | 6.19^***^ | 6.11^***^ |
|  | (70.42) | (69.86) | (22.26) | (22.34) |
| N | 263120 | 263120 | 46478 | 46478 |
| N  clust | 60766 | 60766 | 29361 | 29361 |
| pr2 | .0738 | .0859 | .122 | .135 |

All effect sizes standardized to 1=1 sd

^*^ *p* < 0.05, ^**^ *p* < 0.01, ^***^ *p* < 01

## Demographics

While the newer literature argues that control variables should only be introduced when these are necessary to determine a causal path (Kohler et al., 2023), which is not the case for our x-centred research design, it can nonetheless make sense to test whether our results are robust to including gender, age, age squared and residency in each of Germany’s 16 federal states. As Table OA4 shows, this does not change the main results, both in the sense that not much more total variation in AfD support is explained through the inclusion of deprivation variables, and in the sense that the bivariate link between immigration-concern and AfD-support remains intact.

Table OA 5: controlling for demographics

|  | (1) |  | (2) |  |
| --- | --- | --- | --- | --- |
|  | m1 |  | m2 |  |
| AfD support |  |  |  |  |
| Concern immi | 10^***^ | (31.54) | 8.22^***^ | (28.93) |
| man | 3.83^***^ | (11.63) | 4.7^***^ | (13.69) |
| age | 1.09^***^ | (4.08) | 1.14^***^ | (6.42) |
| age # age | .999^***^ | (-6.38) | .998^***^ | (-8.55) |
| [1] Schleswig-Holstein | 1 | (.) | 1 | (.) |
| [2] Hamburg | .197^*^ | (-2.47) | .234^*^ | (-2.36) |
| [3] Lower Saxony | .696 | (-0.97) | .605 | (-1.41) |
| [4] Bremen | .477 | (-0.96) | .373 | (-1.30) |
| [5] North-Rhine-Westfalia | .456^*^ | (-2.27) | .419^**^ | (-2.62) |
| [6] Hessen | .79 | (-0.60) | .853 | (-0.42) |
| [7] Rheinland-Pfalz | 1.18 | (0.41) | 1.04 | (0.11) |
| [8] Baden-Wuerttemberg | .624 | (-1.28) | .73 | (-0.90) |
| [9] Bavaria | .995 | (-0.01) | 1.03 | (0.07) |
| [10] Saarland | .502 | (-0.88) | .412 | (-1.17) |
| [11] Berlin | .916 | (-0.21) | 1.01 | (0.02) |
| [12] Brandenburg | 7.63^***^ | (5.45) | 7.58^***^ | (5.52) |
| [13] Mecklenburg-Vorpommern | 4.55^***^ | (3.40) | 4.6^***^ | (3.50) |
| [14] Saxony | 20.6^***^ | (8.59) | 21.6^***^ | (8.88) |
| [15] Saxony-Anhalt | 2.71^*^ | (2.43) | 2.43^*^ | (2.21) |
| [16] Thuringia | 10.5^***^ | (6.22) | 10.4^***^ | (6.27) |
| Education |  |  | .445^***^ | (-11.84) |
| Perc hh inc |  |  | .736^***^ | (-5.38) |
| Satisf hh inc |  |  | .782^***^ | (-3.49) |
| Satisf pers inc |  |  | .943 | (-0.81) |
| Satisf life |  |  | .841^***^ | (-4.13) |
| Concern finan |  |  | .931 | (-1.52) |
| Concern econ |  |  | 1.46^***^ | (8.68) |
| / |  |  |  |  |
| lnsig2u | 21^***^ | (55.43) | 19.3^***^ | (56.64) |
| N | 86097 |  | 86097 |  |
| N_clust | 27074 |  | 27074 |  |
| pr2 | .137 |  | .162 |  |

All effect sizes standardized to 1=1 sd

^*^ *p* < 0.05, ^**^ *p* < 0.01, ^***^ *p* < 01

## Other variables to explain AfD-support

We have tested whether deprivation explains AfD-support, based on theoretical considerations of the literature above. An alternative to this is a theoretically agnostic data-mining approach, which instead tests whether *any* variable explains AfD support in addition to what immigration-concern explains alone. We use this approach, by employing all SOEP-variables that could conceivably explain AfD-support. The full list of the 257 variables that we have used is listed in in Table OA5, which also shows how much variance of AfD support each explains when used in conjunction with worries about immigration. We have used the German original of each variable’s label when a translation was unavailable in the SOEP.

Table OA 6: Explained variance after adding variable to immigration-concern

| Variable |  |
| --- | --- |
| [de] Einfluss von Fluechtlingen auf Deutschland als Lebensort | 0.37 |
| Gegen Covid19 geimpft | 0.35 |
| [de] Einfluss von Fluechtlingen auf das kulturelle Leben | 0.35 |
| [de] Europa verbunden | 0.35 |
| [de] Einfluss von Fluechtlingen auf die Wirtschaft | 0.34 |
| [de] Zustrom von Fluechtlingen langfristig mehr Risiko oder Chance | 0.34 |
| [de] Es ist gut dass der Gesetzgeber berücksichtigt dass sich nicht alle Mensc | 0.31 |
| [de] Zustrom von Fluechtlingen kurzfristig mehr Risiko oder Chance | 0.31 |
| [de] Häufigkeit Diskriminierung letzten beiden Jahre: Politische Einstellung | 0.3 |
| [de] Häufigkeit Lesen von (Tages-)Zeitungen (auch ePaper) | 0.3 |
| [de] Dass sich nicht alle Menschen eindeutig mit ihrem biologischen Geschlecht i | 0.3 |
| [de] Schulen und Kindergärten sollten Kinder zu Aktivitäten anregen die nicht t | 0.3 |
| [de] Dass sich gleichgeschlechtliche Paare Kinder wünschen sollte in unserer Ge | 0.29 |
| [de] Einschätzung einwirken auf öffentl. Entscheidungen: Auf kommunaler Ebene | 0.29 |
| [de] Sorgen dass Sie mit dem technischen Fortschritt nicht mithalten können | 0.29 |
| [de] Sorgen dass Ihre beruflichen Qualifikationen abgewertet werden | 0.29 |
| [de] Sorgen dass Berufliches und Privates nicht gut vereinbart werden können | 0.29 |
| [de] Häufigkeit Nutzung sozialer Netzwerke | 0.28 |
| [de] Häufigkeit Lesen von Büchern (auch eBooks) | 0.28 |
| [de] Es ist ganz natürlich dass es auch Menschen gibt die mit männlichen und we | 0.28 |
| [de] Einschätzung einwirken auf öffentl. Entscheidungen: Auf regionaler Ebene | 0.28 |
| [de] Einschätzung einwirken auf öffentl. Entscheidungen: Auf Landesebene | 0.28 |
| [de] Einschätzung einwirken auf öffentl. Entscheidungen: Auf Bundesebene | 0.28 |
| Essentrinken gehen | 0.28 |
| [de] Letzte 12 Monate: Friseure Bars Restaurants etc. besucht | 0.28 |
| Political attitude | 0.27 |
| [de] Häufigkeit Diskriminierung letzten beiden Jahre: Alter | 0.27 |
| [de] Häufigkeit Diskriminierung letzten beiden Jahre: Geschlecht | 0.27 |
| [de] Häufigkeit Diskriminierung letzten beiden Jahre: Einkommen | 0.27 |
| [de] Häufigkeit Diskriminierung letzten beiden Jahre: Bildungsgrad | 0.27 |
| [de] Häufigkeit Diskriminierung letzten beiden Jahre: Sexuelle Orientierung | 0.27 |
| [de] Häufigkeit Diskriminierung letzten beiden Jahre: Religion / Weltanschauung | 0.27 |
| [de] Häufigkeit Diskriminierung letzten beiden Jahre: Rassistische Gründe / (eth | 0.27 |
| [de] Häufigkeit Diskriminierung letzten beiden Jahre: Behinderung | 0.27 |
| [de] Häufigkeit Diskriminierung letzten beiden Jahre: Krankheit | 0.27 |
| [de] Häufigkeit Diskriminierung letzten beiden Jahre: Aus einem anderen Grund | 0.27 |
| [de] Häufigkeit Reparaturen Haus Wohnung oder Fahrzeugen / Gartenarbeit / Fahrz | 0.27 |
| [de] Häufigkeit Einfach nichts tun / abhängen / träumen | 0.27 |
| [de] Die Zuordnung zu einem Geschlecht ist für die Entwicklung eines Menschen ni | 0.27 |
| [de] Es ist gerecht wenn Personen die hart arbeiten mehr verdienen als andere | 0.27 |
| [de] Es ist gerecht wenn Personen die aus angesehenen Familien stammen dadurc | 0.27 |
| [de] Eine Gesellschaft ist gerecht wenn sie sich um die Schwachen und Hilfsbedü | 0.27 |
| [de] Es ist gerecht wenn Einkommen und Vermögen in unserer Gesellschaft an alle | 0.27 |
| [de] Einschätzung einwirken auf öffentl. Entscheidungen: Auf internationaler Ebe | 0.27 |
| Frequency of being lonely in the last 4 weeks | 0.27 |
| Besuche NachbarnFreunde | 0.27 |
| Besuche FamilieVerwandte | 0.27 |
| AusfluegeKurzreisen | 0.27 |
| Fernsehen Video | 0.27 |
| Besuch von Sportveranstaltung | 0.27 |
| Worried About Hostility To Foreigners | 0.27 |
| Most People Are Exploitive Fair | 0.26 |
| [de] Bruttoverdienst gerecht (ja/nein) [2017 2019 2021] | 0.26 |
| [de] Nettoverdienst gerecht (ja/nein) [2017-2019 2021] | 0.26 |
| [de] Unterstuetzung Fluechtlinge zukuenftig: Spenden | 0.26 |
| Benoetigte Ausbildung [harmonisiert] | 0.25 |
| Bei jetzigem Arbeitgeber beschaeftigt [harmonisiert] | 0.25 |
| worried about consequences from climate change | 0.25 |
| Satisfaction With Social Security | 0.25 |
| Confident About Future | 0.25 |
| On The Whole Trust People | 0.25 |
| I think it is good that marriages between two women or two men are legally recog | 0.25 |
| [de] Gut fuer Gesellschaft wenn Transgender-Personen als normal anerkannt werde | 0.25 |
| Most people are basically losers | 0.25 |
| [de] Unterstuetzung Fluechtlinge letztes Jahr: Spenden | 0.25 |
| Tätigkeit entspricht Beruf [harmonisiert] | 0.24 |
| Berufliche Stellung derzeit [harmonisiert] | 0.24 |
| Finanz. Absicherung Alter | 0.24 |
| [de] Kirche Religion [harmonisiert] | 0.24 |
| Angemessenes gehalt | 0.24 |
| Nettoverdienst letzten Monat [harmonisiert] | 0.24 |
| Wie oft Fleisch | 0.24 |
| Wie oft Gefluegel | 0.24 |
| Worried About global terrorism | 0.24 |
| Worried About Job Security | 0.24 |
| [de] Geld gespendet | 0.24 |
| Satisfaction With Standard Of Living | 0.24 |
| Satisfaction With Life A Year From Now | 0.24 |
| Satisfaction With Work | 0.24 |
| Satisfaction With Life Five Years From Now | 0.24 |
| Do Not Enjoy Work | 0.24 |
| Nowadays Can't Trust Anyone | 0.24 |
| Caution Towards Foreigners | 0.24 |
| Most People Are Helpful Act In Own Interest | 0.24 |
| Personal Patience | 0.24 |
| [de] Gleichgeschlechtliches Paar kann Kind genauso gut grossziehen | 0.24 |
| I deserve to be seen as a great personality | 0.24 |
| I want my rivals to fail | 0.24 |
| Befristet/unbefristet [harmonisiert] | 0.23 |
| Finanz. Absicherung Krankheit | 0.23 |
| Finanz. Absicherung Arbeitslosigk. | 0.23 |
| Finanz. Absicherung Plegebeduerftigk. | 0.23 |
| Krankgemeldet ueber 6 Wochen Vorjahr [harmonisiert] | 0.23 |
| Zeit-/Leiharbeit [harmonisiert] | 0.23 |
| Arbeit unter Zeitdruck | 0.23 |
| Gedanken an Arbeitsprobleme beim Aufwachen | 0.23 |
| Abschalten von der Arbeit fällt leicht | 0.23 |
| Aufopfern für Beruf | 0.23 |
| Arbeit geht abends im Kopf rum | 0.23 |
| Schlafprobleme wegen Arbeit | 0.23 |
| Schlechte aufstiegschancen | 0.23 |
| Wie viel Alkohol | 0.23 |
| Wie oft Fisch | 0.23 |
| Vegetarische oder vegane Ernaehrung | 0.23 |
| Worried About Environment | 0.23 |
| Satisfaction With Child Care | 0.23 |
| Lonely | 0.23 |
| Barely Able To Cope With Things | 0.23 |
| Am sometimes too coarse with others | 0.23 |
| Personal Impulsivness | 0.23 |
| Worried About Own Retirement Pension | 0.23 |
| [de] Beschwerden: Wenig Interesse / Freude an Tätigkeiten | 0.23 |
| [de] Beschwerden: Niedergeschlagenheit | 0.23 |
| [de] Beschwerden: Nervosität | 0.23 |
| [de] Beschwerden: Sorgen nicht stoppen können | 0.23 |
| [de] Alleinstehender Elternteil kann Kind genauso gut grossziehen wie beide zusa | 0.23 |
| Being a very special person gives me a lot of strength | 0.23 |
| [de] Mit meinen Beitraegen schaffe ich es im Mittelpunkt zu stehen | 0.23 |
| I react with annoyance if another person steals the show from me | 0.23 |
| [de] Locus of Control:Beeinflussung soz. Verh. durch Engagement [2005 2010 201 | 0.23 |
| [de] Unterstuetzung Fluechtlinge zukuenftig: Arbeit vor Ort mit Fluechtlingen | 0.23 |
| Erwerbsstatus [harmonisiert] | 0.22 |
| Familienstand [harmonisiert] | 0.22 |
| Lebensgemeinschaft [harmonisiert] | 0.22 |
| Sexuelle Orientierung [harmonisiert] | 0.22 |
| VB: Besitz Kraftfahrzeug | 0.22 |
| Anzahl enge Freunde | 0.22 |
| Ausgeglichen letzten 4 Wochen | 0.22 |
| Rauchen gegenwaertig [harmonisiert] | 0.22 |
| Wie oft Alkohol | 0.22 |
| Worried About Peace | 0.22 |
| Satisfaction With Household Income | 0.22 |
| Satisfaction With Personal Income | 0.22 |
| Frequency of being angry in the last 4 weeks | 0.22 |
| I Take Revenge If I Suffer Serious Wrong | 0.22 |
| Payback: Put someone in Difficult Position | 0.22 |
| when Offended Offend Back | 0.22 |
| Forgive and Forget | 0.22 |
| Thorough worker | 0.22 |
| Am communicative | 0.22 |
| Am original | 0.22 |
| Worry a lot | 0.22 |
| Able to forgive | 0.22 |
| Tend to be lazy | 0.22 |
| Am sociable | 0.22 |
| Value artistic experiences | 0.22 |
| Somewhat nervous | 0.22 |
| Carry out tasks efficiently | 0.22 |
| Reserved | 0.22 |
| Friendly with others | 0.22 |
| Have lively imagination | 0.22 |
| Deal well with stress | 0.22 |
| Attitude towards future | 0.22 |
| inquisitive | 0.22 |
| Activities Useful And Valuable | 0.22 |
| Worried About Cohesion in Society | 0.22 |
| [de] Wichtigkeit: Der Glaube die Religion [harmonisiert] | 0.22 |
| [de] Locus of Control:Mein Lebenslauf haengt von mir ab [2005 2010 2015-2016 | 0.22 |
| [de] Locus of Control:Erfahr.dass andere mein Leben bestimmen [2005 2010 2015 | 0.22 |
| Kirchgang Besuch religioeser Veranstaltungen [harmonisiert] | 0.22 |
| [de] Unterstuetzung Fluechtlinge letztes Jahr: Arbeit vor Ort mit Fluechtlingen | 0.22 |
| [de] Unterstuetzung Fluechtlinge letztes Jahr: Teilnahme Demonstration | 0.22 |
| [de] Unterstuetzung Fluechtlinge zukuenftig: Teilnahme Demonstration | 0.22 |
| Eile Zeitdruck letzten 4 Wochen | 0.21 |
| Niedergeschlagen letzten 4 Wochen | 0.21 |
| Energie letzten 4 Wochen | 0.21 |
| Koerperliche Schmerzen letzten 4 Wochen | 0.21 |
| Weniger geschafft wegen körperlicher Probleme | 0.21 |
| Inhaltliche Einschränkung wegen körperlicher Probleme | 0.21 |
| Weniger geschafft wegen seelischer Probleme | 0.21 |
| Weniger geschafft wegen seelischer Probleme | 0.21 |
| Einschränkung sozialer Kontakte wegen Gesundheit | 0.21 |
| Leiden unter chronischen Krankheiten | 0.21 |
| Interesse fuer Politik | 0.21 |
| Worried About Economic Development | 0.21 |
| Worried About Finances | 0.21 |
| Worried About Own Health | 0.21 |
| worried about crime in germany | 0.21 |
| Importance: To Have Success In The job | 0.21 |
| Satisfaction With Health | 0.21 |
| satisfaction with sleep | 0.21 |
| Satisfaction With Housework | 0.21 |
| Satisfaction With Dwelling | 0.21 |
| Satisfaction With Amount Of Leisure Time | 0.21 |
| satisfaction with family life | 0.21 |
| Current Life Satisfaction | 0.21 |
| Frequency of being worried in the last 4 weeks | 0.21 |
| Frequency of being happy in the last 4 weeks | 0.21 |
| Frequency of being sad in the last 4 weeks | 0.21 |
| [de] Selbsteinschaetzung Risikobereitschaft [harmonisiert] | 0.21 |
| I Return Favors | 0.21 |
| Go Out Of My Way To Help Somebody | 0.21 |
| Undergo Costs to Help Somebody Who Helped Before | 0.21 |
| When Feelings Hurt Get Over it Fast | 0.21 |
| When Somebody Wronged Me I Think of it a While | 0.21 |
| Bear Grudges | 0.21 |
| Positive Attitude Toward Myself | 0.21 |
| [de] Mitglied in Gewerkschaft [harmonisiert] | 0.21 |
| [de] Mitglied in Berufsverband [harmonisiert] | 0.21 |
| [de] Locus of Control:Habe nicht das erreichtwas ich verdiene [2005 2010 2015 | 0.21 |
| [de] Locus of Control:Was man erreicht haengt von Glueck ab [2005 2010 2015-20 | 0.21 |
| [de] Locus of Control:Erfolg ist hart erarbeitet [2005 2010 2015-2016 2020] | 0.21 |
| [de] Locus of Control:Zweifle bei Schwierigkeiten an Faehigkt. [2005 2010 2015 | 0.21 |
| [de] Locus of Control:Moeglichk. von soz. Umstaenden bestimmt [2005 2010 2015- | 0.21 |
| [de] Locus of Control:Faehigkeiten wichtiger als Anstrengung [2005 2010 2015-2 | 0.21 |
| [de] Locus of Control:Wenig Kontrolle ueber Dinge in m. Leben [2005 2010 2015- | 0.21 |
| Besuch von Oper klass. Konzerte Theater Ausstellungen [harmonisiert] | 0.21 |
| Kuenstlerische und musische Taetigkeiten [harmonisiert] | 0.21 |
| Ehrenamtliche Taetigkeit in Vereinen Verbaenden … [harmonisiert] | 0.21 |
| Isolated from others | 0.21 |
| Arbeitsintensitaet Veraenderung | 0.2 |
| Art der Krankenversicherung [1999-2020] | 0.2 |
| Importance: To Be Able To Afford Sth. | 0.2 |
| Importance: To help Others | 0.2 |
| Importance: To Fulfil Once Potential | 0.2 |
| Importance: To Have An Own House | 0.2 |
| Importance: To have a happy marriage | 0.2 |
| Importance: To have children | 0.2 |
| Importance: To be socially and politically active | 0.2 |
| Importance: To be able to travel and see the world | 0.2 |
| Satisfaction With School Education and Vocational Retraining | 0.2 |
| [de] Nachdenken ueber geld | 0.2 |
| Aktiver Sport [harmonisiert] | 0.2 |
| Beteilig. Parteien Kommunalpolitik Buergerinitiativen [harmonisiert] | 0.2 |
| Ties To Local Area | 0.2 |
| Visited Foreigners Previous Yr | 0.2 |
| Received Visits from Foreigners Previous Yr | 0.2 |
| Contact To Friends Relatives Abroad | 0.2 |
| Stays >3 Months Abroad | 0.2 |
| Both Parents Born In Germany | 0.2 |
| That you lack companionship | 0.2 |
| Feel left out | 0.2 |
| Worried Future of the European Union | 0.2 |
| worried about stability of financial markets | 0.19 |
| Besuch von KinoPopJazz KonzerteDisco [harmonisiert] | 0.19 |
| Visited Germans Previous Yr | 0.19 |
| Received Visits from Germans Previous Yr | 0.19 |
| gilft of 100 EUR give away a contingent | 0.18 |
| [de] Zufriedenheit Einkommen (letzte 10 Jahre) | 0.18 |
| gift of 100 EUR save a contingent | 0.17 |
| gift of 100 EUR spend a contingent | 0.17 |
| Satisfaction With Area You Live In | 0.17 |
| Satisfaction With Social Life | 0.17 |
| [de] Zufriedenheit Lebensverhaeltnisse (letzte 10 Jahre) | 0.17 |
| [de] Zufriedenheit Gesundheit (letzte 10 Jahre) | 0.17 |
| Satisfaction Realization Social Equity | 0.16 |
| Geselligkeit m. Freund. Verwandt. [harmonisiert] | 0.16 |
| Mithelfen bei Freund. Verwandt. [harmonisiert] | 0.16 |
| Chancen gleichwertige Stelle zu finden | 0.13 |
| Willingness To Take Risks In Occupation | 0.086 |
| Willingness To Take Risks In Leisure And Sports | 0.076 |
| Willingness To Take Risks In Financial Matters | 0.075 |
| Gesundheitsbewusste Ernaehrung | 0.074 |
| Willingness To Take Risks While Driving | 0.074 |
| Willingness To Take Risks In Trusting Other People | 0.074 |
| Willingness To Take Health Risks | 0.073 |
| Lebenslanges Lernen | 0.072 |

Are workers closer to the AfD irrespective of concern about immigration?

We used the variable pgstib from SOEP’s dataset pgen to code the following occupational categories as workers: [200] Worker, no further information; [210] Untrained Worker; [220] Semi-Trained Worker; [230] Trained Worker; [240] Foreman, group leader; [250] Foreman; [310] Blue-collar worker in agriculture; [320] Skilled worker in agriculture.

|  | (1) |  | (2) |  |
| --- | --- | --- | --- | --- |
|  | m1 |  | m3 |  |
| AfD support |  |  |  |  |
| Concern immi | 11.2^***^ | (36.75) | 10.9^***^ | (36.68) |
| worker |  |  | 2.68^***^ | (8.41) |
| / |  |  |  |  |
| lnsig2u | 21.3^***^ | (72.28) | 20.8^***^ | (70.06) |
| N | 99918 |  | 99918 |  |
| N_clust | 30713 |  | 30713 |  |
| pr2 | .0822 |  | .085 |  |

All effect sizes standardized to 1=1 sd, except that worker is 0/1

^*^ *p* < 0.05, ^**^ *p* < 0.01, ^***^ *p* < 0.001

Kohler, U., Class, F., & Sawert, T. (2023). Control Variable Selection in Applied Quantitative Sociology: A Critical Review. *European Sociological Review*. <https://doi.org/10.1093/esr/jcac078>

## Stata code to reproduce everything

global working ""

cd $working

set scheme cblind1

set processor 6

label language EN

use "soepv41/pl.dta", clear

sort pid syear

merge 1:1 pid syear using "soepv41/ppathl.dta", keepusing(phrf)

drop if _merge ==2 // useless if only in ppathl

drop _merge

merge 1:1 pid syear using "soepv41/pequiv.dta", keepusing(i11102 i11110 d11106 x11104ll d11102ll d11101 l11101)

drop if _merge ==2 // useless if only in pequiv

drop _merge

recode d11102ll (2=0 "Female") (1=1 "Male") (else=.), gen("man")

generate age =d11101 if d11101>=0

clonevar state=l11101

merge 1:1 pid syear using "soepv41/pgen.dta", keepusing(pgisced97 pgstib)

drop if _merge ==2 // useless if only in pgen

gen worker_pgstib = inrange(pgstib,200,320)

tab pgstib if worker_pgstib == 0

label language EN

label var worker_pgstib "worker based on occupational classification"

label define worker_pgstib 1 "worker" 0 "not worker"

label values worker_pgstib worker_pgstib

foreach var of varlist i11102 i11110 d11106 {

replace `var'=. if `var'<0

sum `var'

}

gen ind_inc=i11110

gen hh_net_equiv_inc=i11102/(d11106^.5)

xtset pid syear

sort pid syear

replace hh_net_equiv_inc=l.hh_net_equiv_inc if l.hh_net_equiv_inc!=. & hh_net_equiv_inc==.

replace hh_net_equiv_inc=l2.hh_net_equiv_inc if l2.hh_net_equiv_inc!=. & hh_net_equiv_inc==.

drop if syear<2013

*AfD variables

recode plh0012_h (27=1) (1/6 9/11 13/17 20 22/24=0) (else=.) , gen(afd_pref_vs_oth)

recode plh0012_h (27/31=1) (-4=.) (else=0) , gen(afd_pref_vs_all_oth)

recode plh0333 (1/6 9/20 22/24 = 0) (27 30 31 = 1) (else=.), gen(afd_vote)

label var afd_pref_vs_all_oth "AfD pref vs all"

label var afd_vote "AfD vote vs establ party"

* drop if ind var unavailable

drop if afd_pref_vs_oth==. & afd_pref_vs_all_oth==. & afd_vote==.

sum afd_pref_vs_oth afd_pref_vs_all_oth afd_vote

keep pid syear plh* plj* *afd* ind_inc hh_net_equiv_inc pgisced97 ind_inc hh_net_equiv_inc phrf i11102 i11110 d11106 x11104ll man age worker_pgstib state // only keep necessary variables

foreach var of varlist plh0155 plh0171 plh0172 plh0175 plh0176 plh0177 plh0178 plh0180 plh0182 plh0032 plh0033 plh0035 plh0036 plh0037 plh0038 plh0040 plh0335 plh0336 plj0046 plj0047 {

* sum `var' if afd_pref_vs_oth==1 // how many cases with AfD pref

di "clonevar" " " `"`: var label `var''"' " " "=" " " "`var'"

}

clonevar zufr_hh_eink = plh0175

clonevar zufr_pers_eink = plh0176

clonevar zufr_wohnung = plh0177

clonevar zufr_freizeit = plh0178

clonevar zufr_fam = plh0180

clonevar zufr_leben = plh0182

clonevar sorgen_allg_wirt_sit = plh0032

clonevar sorgen_eig_wirt_sit = plh0033

clonevar sorgen_eig_gesund = plh0035

clonevar sorgen_umwelt = plh0036

clonevar sorgen_klima = plh0037

clonevar sorgen_frieden = plh0038

clonevar sorgen_kriminal = plh0040

clonevar sorgen_zuwanderung = plj0046

clonevar sorgen_auslaenderf = plj0047 // simply doubles sorgen_zuwanderung

clonevar zufr_demokr=plh0152_v2

drop if sorgen_zuwanderung<0 // makes no sense if important variable not available

foreach var of varlist * {

capture replace `var'=. if `var'<0

}

foreach var of varlist sorgen* {

revrs `var', replace

}

label values sorgen_* .

egen perc_ind_inc=xtile(ind_inc), n(100) by(syear)

egen perc_hh_net_equiv_inc=xtile(hh_net_equiv_inc), n(100) by(syear)

replace pgisced97=. if pgisced97==0

missings dropvars *, force

foreach var of varlist zufr_* sorgen_* perc_ind_inc perc_hh_net_equiv_inc pgisced97 {

sum `var'

egen sd_`var'=std(`var')

sum sd_`var'

}

foreach var of varlist sd_* {

disp "`var' " "`var' "

}

label var afd_pref_vs_oth "AfD support"

label var sorgen_zuwanderung "Concern immi"

label var zufr_hh_eink "Satisf hh inc"

label var zufr_pers_eink "Satisf pers inc"

label var zufr_leben "Satisf life"

label var perc_hh_net_equiv_inc "Percentile hh net equiv inc"

label var pgisced97 "Education PGISCED 97 scale"

label var sorgen_eig_wirt_sit "Concern own finan"

label var sorgen_allg_wirt_sit "Concern Ger economy"

label var zufr_demokr "Satisfaction democracy"

label var sd_zufr_hh_eink "Satisf hh inc"

label var sd_zufr_pers_eink "Satisf pers inc"

label var sd_zufr_wohnung "Satisf flat"

label var sd_zufr_freizeit "Satisf leisure"

label var sd_zufr_fam "Satisf family"

label var sd_zufr_leben "Satisf life"

label var sd_sorgen_allg_wirt_sit "Concern econ"

label var sd_sorgen_eig_wirt_sit "Concern finan"

label var sd_sorgen_eig_gesund "Concern health"

label var sd_sorgen_umwelt "Concern environm"

label var sd_sorgen_klima "Concern climate"

label var sd_sorgen_frieden "Concern peace"

label var sd_sorgen_kriminal "Concern criminal"

label var sd_sorgen_zuwanderung "Concern immi"

label var sd_sorgen_auslaenderf "Concern xenoph"

label var sd_perc_hh_net_equiv_inc "Perc hh inc"

label var sd_pgisced97 "Education"

*ssc install r2_mz

* so that all effects are positive

revrs sd_sorgen_auslaenderf sd_sorgen_klima sd_sorgen_frieden sd_zufr_hh_eink sd_zufr_wohnung sd_zufr_pers_eink sd_sorgen_umwelt sd_zufr_leben sd_perc_ind_inc sd_perc_hh_net_equiv_inc sd_pgisced97 sd_zufr_demokr

label var revsd_sorgen_auslaenderf "unw xenophobia"

label var revsd_sorgen_klima "unw climate"

label var revsd_sorgen_frieden "un peace"

label var revsd_zufr_hh_eink "unsat hh inc "

label var revsd_zufr_wohnung "unsat flat"

label var revsd_zufr_pers_eink "unsat pers inc"

label var revsd_sorgen_umwelt "unw environm"

label var revsd_zufr_leben "unsat life"

label var revsd_perc_ind_inc "low pers inc"

label var revsd_perc_hh_net_equiv_inc "low hh net inc"

label var revsd_pgisced97 "low edu"

label var revsd_zufr_demokr "unsat democr"

format afd_pref_vs_oth sorgen_zuwanderung zufr_hh_eink zufr_pers_eink zufr_leben perc_hh_net_equiv_inc pgisced97 sorgen_eig_wirt_sit sorgen_allg_wirt_sit zufr_demokr %9.1f

sort pid syear

bysort pid (syear): gen last_weight = phrf[_N]

global working "/"

cd $working

save afd.dta, replace

use afd.dta, clear

sum syear

global max_year = r(max)

disp $max_year

********************************************************************************

*descriptive

*ssc install fsum

* TableOA1

foreach var of varlist afd_pref_vs_oth sd_sorgen_zuwanderung ///

sd_zufr_hh_eink sd_zufr_pers_eink sd_zufr_leben sd_perc_hh_net_equiv_inc sd_pgisced97 ///

sd_sorgen_eig_wirt_sit sd_sorgen_allg_wirt_sit {

drop if `var'==.

}

fsum afd_pref_vs_oth sorgen_zuwanderung zufr_hh_eink zufr_pers_eink zufr_leben perc_hh_net_equiv_inc pgisced97 sorgen_eig_wirt_sit sorgen_allg_wirt_sit, stat(n mean sd min max) format(%8.2f) uselabel

********************************************************************************

*bivariate

pwcorr afd_pref_vs_oth sd_sorgen_zuwanderung revsd_pgisced97 revsd_perc_hh_net_equiv_inc revsd_zufr_hh_eink revsd_zufr_pers_eink revsd_zufr_leben sd_sorgen_eig_wirt_sit sd_sorgen_allg_wirt_sit, star(.01)

*annually

estimates clear

eststo clear

use $working/afd.dta, clear

keep if afd_pref_vs_oth!=.

* afd is one-issue party in almost every year

foreach num of numlist 2014/2024 {

preserve

keep if syear==`num'

tab syear

eststo syear`num': logit afd_pref_vs_oth sd_sorgen_zuwanderung sd_zufr_hh_eink sd_zufr_pers_eink sd_zufr_leben sd_perc_hh_net_equiv_inc sd_pgisced97 sd_sorgen_eig_wirt_sit sd_sorgen_allg_wirt_sit [pweight=phrf], vce(robust)

margins, dydx(*) atmeans post

estimates store margins_`num'

coefplot margins_`num', rescale(100) xline(0) drop(_cons) mlabel mlabsize(medsmall) mlabformat(%3.1f) mlabpos(2) title(`num') saving(syear`num'.gph, replace) sort labels xlabel(-2(1)5,nogrid) grid(none)

restore

}

esttab syear* using OA2.rtf, replace stat(N r2_p) mtitle label eform note("All effect sizes standardized to 1=1 sd") nogaps b(%9.3g)

shell open OA2.rtf

graph combine syear2014.gph syear2015.gph syear2016.gph syear2017.gph syear2018.gph syear2019.gph syear2020.gph syear2021.gph syear2022.gph syear2023.gph syear2024.gph, scale(1) imargin(zero) col(2) note("All effect sizes average marginal effects of % change AfD sympathy based on variables standardized to sd=1") ysize(8)

graph export Figure1.png, replace

sum afd_pref_vs_oth if syear==2014 [aweight=phrf]

sum afd_pref_vs_oth if syear==$max_year [aweight=last_weight]

********************************************************************************

cd $working/

use $working/afd.dta, clear

foreach var of varlist afd_pref_vs_oth sd_sorgen_zuwanderung ///

sd_zufr_hh_eink sd_zufr_pers_eink sd_zufr_leben sd_perc_hh_net_equiv_inc sd_pgisced97 ///

sd_sorgen_eig_wirt_sit sd_sorgen_allg_wirt_sit {

drop if `var'==.

}

eststo clear

estimates clear

* null

xtlogit afd_pref_vs_oth, or vce(robust)

local ll0 = e(ll)

* concerned immigration

xtlogit afd_pref_vs_oth sd_sorgen_zuwanderung, or vce(robust)

estimates store Concern_immigration

local ll = e(ll)

estadd scalar pr2 = (`ll0' - `ll')/`ll0'

* actual deprivation

xtlogit afd_pref_vs_oth sd_sorgen_zuwanderung ///

sd_pgisced97 sd_perc_hh_net_equiv_inc sd_zufr_hh_eink sd_zufr_pers_eink sd_zufr_leben ///

, or vce(robust)

estimates store Objective_deprivation

local ll = e(ll)

estadd scalar pr2 = (`ll0' - `ll')/`ll0'

* feared deprivation

xtlogit afd_pref_vs_oth sd_sorgen_zuwanderung ///

sd_sorgen_eig_wirt_sit sd_sorgen_allg_wirt_sit ///

, or vce(robust)

estimates store Feared_deprivation

local ll = e(ll)

estadd scalar pr2 = (`ll0' - `ll')/`ll0'

* all

xtlogit afd_pref_vs_oth sd_sorgen_zuwanderung ///

sd_pgisced97 sd_perc_hh_net_equiv_inc sd_zufr_hh_eink sd_zufr_pers_eink sd_zufr_leben ///

sd_sorgen_eig_wirt_sit sd_sorgen_allg_wirt_sit ///

, or vce(robust)

estimates store All

local ll = e(ll)

estadd scalar pr2 = (`ll0' - `ll')/`ll0'

* last year's deprivation

xtlogit afd_pref_vs_oth sd_sorgen_zuwanderung ///

sd_pgisced97 l.sd_perc_hh_net_equiv_inc l.sd_zufr_hh_eink l.sd_zufr_pers_eink l.sd_zufr_leben ///

l.sd_sorgen_eig_wirt_sit l.sd_sorgen_allg_wirt_sit ///

, or vce(robust)

estimates store Lagged_deprivation

local ll = e(ll)

estadd scalar pr2 = (`ll0' - `ll')/`ll0'

* Interactions

xtlogit afd_pref_vs_oth sd_sorgen_zuwanderung ///

sd_pgisced97 sd_perc_hh_net_equiv_inc sd_zufr_hh_eink sd_zufr_pers_eink sd_zufr_leben sd_sorgen_eig_wirt_sit sd_sorgen_allg_wirt_sit ///

c.sd_pgisced97#c.sd_sorgen_zuwanderung c.sd_perc_hh_net_equiv_inc#c.sd_sorgen_zuwanderung c.sd_zufr_hh_eink#c.sd_sorgen_zuwanderung c.sd_zufr_pers_eink#c.sd_sorgen_zuwanderung c.sd_zufr_leben#c.sd_sorgen_zuwanderung c.sd_sorgen_eig_wirt_sit#c.sd_sorgen_zuwanderung c.sd_sorgen_allg_wirt_sit#c.sd_sorgen_zuwanderung ///

, or vce(robust)

estimates store Interactions

local ll = e(ll)

estadd scalar pr2 = (`ll0' - `ll')/`ll0'

esttab Concern_immigration Objective_deprivation Feared_deprivation All Lagged_deprivation Interactions using Table1.rtf, replace stat(N N_clust ll aic bic pr2) mtitle label eform note("All effect sizes standardized to 1=1 sd; Table shows odds ratios based on Stata xtlogit re procedure with robust standard errors") nogaps b(%9.4g)

shell open Table1.rtf

* compare Model 4 effects

estimates restore All

margins, dydx(*) post atmeans

estimates store margins

estimates restore margins

coefplot margins, mlabel mlabformat(%9.2g) mlabpos(2) sort(1:) xline(0) title() note("All effect sizes average marginal effects of % change AfD support, based on variables standardized to sd=1") saving(Figure2.gph, replace) legend(col(1)) legend(off) scale(1.3) xlabel(,nogrid) grid(none) rescale(100)

graph export Figure2.png, replace

********************************************************************************

* margins for guy who is perfect but concerned about immigration and contrary

cd $working/

use afd.dta, clear

xtlogit afd_pref_vs_oth sorgen_zuwanderung pgisced97 perc_hh_net_equiv_inc zufr_hh_eink zufr_pers_eink zufr_leben sorgen_eig_wirt_sit sorgen_allg_wirt_sit , or vce(robust)

margins, at(sorgen_zuwanderung==3 pgisced97==6 perc_hh_net_equiv_inc==100 zufr_hh_eink=10 zufr_pers_eink==10 zufr_leben==10 sorgen_eig_wirt_sit==1 sorgen_allg_wirt_sit==1) ///

at(sorgen_zuwanderung==1 pgisced97==1 perc_hh_net_equiv_inc==1 zufr_hh_eink=0 zufr_pers_eink==0 zufr_leben==0 sorgen_eig_wirt_sit==3 sorgen_allg_wirt_sit==3) atmeans post

estimates store margins

* compare indidual here...

coefplot margins, ylabel(1 "immigr-unconc depriv loser" 2 "immigrat-concerned winner", angle(90) alternate) mlabel mlabformat(%9.1f) mlabposition(2) xtitle(Probability AfD-support) scale(1.3) mlabsize(small) xlabel(,nogrid) grid(none) ysize(1) xsize(1.5) rescale(100)

graph export Figure3.png, replace

********************************************************************************

* FE

cd $working/

use afd.dta, clear

foreach var of varlist afd_pref_vs_oth sd_sorgen_zuwanderung ///

sd_zufr_hh_eink sd_zufr_pers_eink sd_zufr_leben sd_perc_hh_net_equiv_inc ///

sd_sorgen_eig_wirt_sit sd_sorgen_allg_wirt_sit {

drop if `var'==.

}

eststo clear

estimates clear

* concerned immigration

xtlogit afd_pref_vs_oth sd_sorgen_zuwanderung, or fe vce(boot)

estimates store Concern_immigration

* actual deprivation

xtlogit afd_pref_vs_oth sd_sorgen_zuwanderung ///

sd_perc_hh_net_equiv_inc sd_zufr_hh_eink sd_zufr_pers_eink sd_zufr_leben ///

, or fe vce(boot)

estimates store Objective_deprivation

* feared deprivation

xtlogit afd_pref_vs_oth sd_sorgen_zuwanderung ///

sd_sorgen_eig_wirt_sit sd_sorgen_allg_wirt_sit ///

, or fe vce(boot)

estimates store Feared_deprivation

* all

xtlogit afd_pref_vs_oth sd_sorgen_zuwanderung ///

sd_perc_hh_net_equiv_inc sd_zufr_hh_eink sd_zufr_pers_eink sd_zufr_leben ///

sd_sorgen_eig_wirt_sit sd_sorgen_allg_wirt_sit ///

, or fe vce(boot)

estimates store All

sort pid syear

* last year's deprivation

xtlogit afd_pref_vs_oth sd_sorgen_zuwanderung ///

l.sd_perc_hh_net_equiv_inc l.sd_zufr_hh_eink l.sd_zufr_pers_eink l.sd_zufr_leben ///

l.sd_sorgen_eig_wirt_sit l.sd_sorgen_allg_wirt_sit ///

, or fe vce(boot)

estimates store Lagged_deprivation

* Interactions

xtlogit afd_pref_vs_oth sd_sorgen_zuwanderung ///

sd_perc_hh_net_equiv_inc sd_zufr_hh_eink sd_zufr_pers_eink sd_zufr_leben sd_sorgen_eig_wirt_sit sd_sorgen_allg_wirt_sit ///

c.sd_perc_hh_net_equiv_inc#c.sd_sorgen_zuwanderung c.sd_zufr_hh_eink#c.sd_sorgen_zuwanderung c.sd_zufr_pers_eink#c.sd_sorgen_zuwanderung c.sd_zufr_leben#c.sd_sorgen_zuwanderung c.sd_sorgen_eig_wirt_sit#c.sd_sorgen_zuwanderung c.sd_sorgen_allg_wirt_sit#c.sd_sorgen_zuwanderung ///

, fe or vce(boot)

estimates store Interactions

esttab Concern_immigration Objective_deprivation Feared_deprivation All Lagged_deprivation Interactions using Table3.rtf, replace stat(N N_clust ll aic bic r2_p) mtitle label eform note("All effect sizes standardized to 1=1 sd, Standard errors based on bootstrapping, as xtlogit fe does not allow robust standard errors") nogaps b(%9.3g)

shell open Table3.rtf

* compare Model 4 effects

estimates restore All

margins, dydx(*) post atmeans

estimates store margins

estimates restore margins

coefplot margins, mlabel mlabformat(%9.2g) mlabpos(2) sort(1:) xline(0) title() note("All effect sizes average marginal effects of % change AfD support, based on variables standardized to sd=1") saving(Figure4.gph, replace) legend(col(1)) legend(off) scale(1.3) xlabel(,nogrid) grid(none) rescale(100)

graph export Figure4.png, replace

********************************************************************************

*State-level AfD effect

cd $working/

use afd.dta, clear

foreach var of varlist afd_pref_vs_oth sd_sorgen_zuwanderung ///

sd_zufr_hh_eink sd_zufr_pers_eink sd_zufr_leben sd_perc_hh_net_equiv_inc sd_pgisced97 ///

sd_sorgen_eig_wirt_sit sd_sorgen_allg_wirt_sit {

drop if `var'==.

}

bysort state syear: egen av_sd_sorgen_zuwanderung = mean(sd_sorgen_zuwanderung)

label var av_sd_sorgen_zuwanderung "Popul concern immi"

egen cstate = group(state syear), label

*collapse av_sd_sorgen_zuwanderung afd_pref_vs_oth sd_sorgen_zuwanderung, by(cstate)

*scatter afd_pref_vs_oth av_sd_sorgen_zuwanderung, mlabel(cstate) msymbol(none) scale(.5)

eststo clear

estimates clear

* null

melogit afd_pref_vs_oth || _all: R.syear || state: , or difficult

local ll0 = e(ll)

* concerned immigration

melogit afd_pref_vs_oth sd_sorgen_zuwanderung av_sd_sorgen_zuwanderung || _all: R.syear || state: , or difficult

estimates store Concern_immigration

local ll = e(ll)

estadd scalar pr2 = (`ll0' - `ll')/`ll0'

* all

melogit afd_pref_vs_oth sd_sorgen_zuwanderung av_sd_sorgen_zuwanderung ///

sd_pgisced97 sd_perc_hh_net_equiv_inc sd_zufr_hh_eink sd_zufr_pers_eink sd_zufr_leben ///

sd_sorgen_eig_wirt_sit sd_sorgen_allg_wirt_sit || _all: R.syear || state: , or difficult

estimates store All

local ll = e(ll)

estadd scalar pr2 = (`ll0' - `ll')/`ll0'

esttab Concern_immigration All using TableOA3.rtf, replace stat(N N_clust pr2) mtitle label eform note("All effect sizes standardized to 1=1 sd") nogaps b(%9.4g)

shell open TableOA3.rtf

* compare Model 4 effects

estimates restore All

margins, dydx(*) post atmeans

estimates store margins

coefplot margins, drop(_cons) mlabel mlabformat(%9.2g) mlabpos(2) sort(1:) xline(0) title() note("All effect sizes average marginal effects of % change AfD sympathy, based on variables standardized to sd=1") saving(X.gph, replace) legend(col(1)) legend(off) scale(1.3) xlabel(,nogrid) grid(none) rescale(100)

graph export FigureOA1.png, replace

********************************************************************************

* other coding of AfD

eststo clear

estimates clear

use afd.dta, clear

foreach var of varlist sd_sorgen_zuwanderung ///

sd_zufr_hh_eink sd_zufr_pers_eink sd_zufr_leben sd_perc_hh_net_equiv_inc sd_pgisced97 ///

sd_sorgen_eig_wirt_sit sd_sorgen_allg_wirt_sit {

drop if `var'==.

}

foreach var of varlist afd_pref_vs_all_oth afd_vote {

preserve

keep if `var'!=.

* null

xtlogit `var', or vce(robust)

local ll0 = e(ll)

* concerned immigration

xtlogit `var' sd_sorgen_zuwanderung, or vce(robust)

estimates store `var'

local ll = e(ll)

estadd scalar pr2 = (`ll0' - `ll')/`ll0'

* all

xtlogit `var' sd_sorgen_zuwanderung ///

sd_pgisced97 sd_perc_hh_net_equiv_inc sd_zufr_hh_eink sd_zufr_pers_eink sd_zufr_leben ///

sd_sorgen_eig_wirt_sit sd_sorgen_allg_wirt_sit ///

, or vce(robust)

estimates store `var'_depriv

local ll = e(ll)

estadd scalar pr2 = (`ll0' - `ll')/`ll0'

restore

}

esttab * using TableOA4.rtf, replace stat(N N_clust pr2) mtitle label eform note("All effect sizes standardized to 1=1 sd") nogaps b(%9.3g)

shell open TableOA4.rtf

* compare effects of immigration-concerns across models

coefplot *, keep(sd_sorgen_zuwanderung) mlabel mlabformat(%3.2g) mlabpos(2) eform title() note("All effect sizes standardized") saving(btw.gph, replace) legend(col(1)) scale(1.2) xlabel(1(1)10) plotlabels("before controls" "after adjusting for deprivation" "after adjusting for feared deprivation" "after adjusting for all types of deprivation") ylabel(1 "Effect of immigration-concern on AfD-support", angle(90))

********************************************************************************

* adjusting for demographics

* other coding of AfD

eststo clear

estimates clear

use afd.dta, clear

foreach var of varlist afd_pref_vs_oth sd_sorgen_zuwanderung ///

sd_zufr_hh_eink sd_zufr_pers_eink sd_zufr_leben sd_perc_hh_net_equiv_inc sd_pgisced97 ///

sd_sorgen_eig_wirt_sit sd_sorgen_allg_wirt_sit man age state {

drop if `var'==.

}

* null

xtlogit afd_pref_vs_oth, or vce(robust)

local ll0 = e(ll)

* concerned immigration

xtlogit afd_pref_vs_oth sd_sorgen_zuwanderung man c.age##c.age i.state, or vce(robust)

estimates store m1

local ll = e(ll)

estadd scalar pr2 = (`ll0' - `ll')/`ll0'

* all

xtlogit afd_pref_vs_oth sd_sorgen_zuwanderung man c.age##c.age i.state ///

sd_pgisced97 sd_perc_hh_net_equiv_inc sd_zufr_hh_eink sd_zufr_pers_eink sd_zufr_leben ///

sd_sorgen_eig_wirt_sit sd_sorgen_allg_wirt_sit ///

, or vce(robust)

estimates store m2

local ll = e(ll)

estadd scalar pr2 = (`ll0' - `ll')/`ll0'

esttab * using TableOA5.rtf, replace stat(N N_clust pr2) mtitle label eform note("All effect sizes standardized to 1=1 sd") nogaps b(%9.3g) wide

shell open TableOA5.rtf

********************************************************************************

* More variables for Online Annex

use afd.dta, clear

merge 1:1 pid syear using "soepv41/pl.dta", keepusing(pl*)

foreach var of varlist afd_pref_vs_oth sd_sorgen_zuwanderung ///

revsd_zufr_hh_eink revsd_zufr_pers_eink revsd_zufr_leben revsd_perc_hh_net_equiv_inc revsd_pgisced97 ///

sd_sorgen_eig_wirt_sit sd_sorgen_allg_wirt_sit {

drop if `var'==.

}

* Loop over the variables to check for the percentage of missing values

foreach var of varlist pl* {

* Calculate the percentage of missing values

capture replace `var'=. if `var'<0

qui count if missing(`var')

local missings = r(N)

local total = _N

local pct_missing = 100 * `missings' / `total'

* Drop the variable if the percentage exceeds the threshold

if `pct_missing' > 90 {

drop `var'

di "Dropped variable: `var' with `pct_missing'% missing values"

}

}

save afd_temp.dta, replace

use afd_temp.dta, clear

eststo clear

estimates clear

global cat_vars "plb0022_h plb0037_h plb0072_h plb0073_h plb0568_h plc0111 plc0112 plc0113 plc0114 pld0131_h pld0132_h pld0298_h plh0258_h"

global metric_vars "plb0024_h plb0036_h plb0041_h plb0112 plb0113 plb0114 plb0115 plb0116 plb0117 plb0443 plb0594 plb0616 plc0014_h plc0552 pld0047 ple0026 ple0027 ple0028 ple0029 ple0030 ple0031 ple0032 ple0033 ple0034 ple0035 ple0036 ple0081_h ple0095 ple0097_v1 ple0177 ple0178 ple0179 ple0180 ple0181 ple0182 ple0200 plg0266 plh0004 plh0007 plh0032 plh0033 plh0034 plh0035 plh0036 plh0037 plh0038 plh0039 plh0040 plh0042 plh0104 plh0105 plh0106 plh0107 plh0108 plh0109 plh0110 plh0111 plh0112 plh0129 plh0134 plh0135 plh0136 plh0155 plh0156 plh0162 plh0164 plh0166 plh0171 plh0172 plh0173 plh0174 plh0175 plh0176 plh0177 plh0178 plh0179 plh0180 plh0182 plh0183 plh0184 plh0185 plh0186 plh0187 plh0188 plh0189 plh0190 plh0191 plh0192 plh0193 plh0194 plh0195 plh0196 plh0197 plh0198 plh0199 plh0201 plh0202 plh0204_h plh0206i01 plh0206i02 plh0206i03 plh0206i04 plh0206i05 plh0206i06 plh0206i07 plh0206i08 plh0206i09 plh0206i10 plh0206i11 plh0212 plh0213 plh0214 plh0215 plh0216 plh0217 plh0218 plh0219 plh0220 plh0221 plh0222 plh0223 plh0224 plh0225 plh0226 plh0244 plh0253 plh0254 plh0255 plh0263_h plh0264_h plh0334 plh0335 plh0336 plh0337_v2 plh0338_v2 plh0339 plh0340 plh0341 plh0342 plh0343_h plh0344 plh0357 plh0358 plh0359 plh0360 plh0361 plh0362 plh0363 plh0364 plh0365 plh0366 plh0377_v2 plh0378_v2 plh0379_v2 plh0380_v2 plh0381_v2 plh0382_v2 plh0383_v2 plh0384_v2 plh0385_v2 plh0386_v2 plh0387i01 plh0387i02 plh0387i03 plh0387i04 plh0387i05 plh0387i06 plh0387i07 plh0387i08 plh0387i09 plh0387i10 plh0387i11 plh0390 plh0391 plh0392 plh0393 plh0394 plh0395i01 plh0395i02 plh0395i03 plh0395i04 plh0395i05 plh0395i06 plh0396i01 plh0396i02 plh0396i03 plh0396i04 plh0397i01 plh0397i02 plh0397i03 plh0397i04 plh0397i05 plh0407 plh0408 plh0409 plh0410 pli0079 pli0080 pli0081 pli0082 pli0083 pli0089 pli0090_h pli0091_h pli0092_h pli0093_h pli0094_h pli0095_h pli0096_h pli0097_h pli0098_h plj0047 plj0043 plj0060 plj0061 plj0062 plj0063 plj0104 plj0105 plj0175 plj0433_h plj0434_h plj0435_h plj0436_h plj0437_h plj0438 plj0439 plj0440 plj0441 plj0442 plj0443 plj0587 plj0588 plj0589 plj0713 plj0723 plj0724"

* categorical vars

foreach var of varlist $cat_vars {

local label: variable label `var' // remove commas from labels

local newlabel = subinstr("`label'", ",", "", .)

label variable `var' "`newlabel'"

egen std_`var'=std(`var')

logit afd_pref_vs_oth sd_sorgen_zuwanderung i.`var', or vce(robust)

estimates store `var'

}

* metric vars

foreach var of varlist $metric_vars {

local label: variable label `var' // remove commas from labels

local newlabel = subinstr("`label'", ",", "", .)

label variable `var' "`newlabel'"

egen std_`var'=std(`var')

label var std_`var' "`: var label `var'' SD"

logit afd_pref_vs_oth sd_sorgen_zuwanderung std_`var', or vce(robust)

estimates store `var'

}

* Use Excel for Online Annex

esttab * using other_vars.csv, replace stat(N r2_p) mtitle label eform note("All effect sizes standardized to 1=1 sd") nogaps b(%9.3g) compress not

shell open -a "Microsoft Excel" "other_vars.csv"

foreach var of varlist plj0435_h ple0200 plj0434_h plj0724 plj0433_h plj0437_h plh0395i01 plj0436_h plh0387i06 plh0392 plh0395i02 plh0395i05 plh0395i06 plh0397i01 plh0408 plh0409 plh0410 plh0390 plh0391 plh0395i03 plh0397i02 plh0397i03 plh0397i04 pli0079 plj0723 plh0004 plh0387i01 plh0387i02 plh0387i03 plh0387i04 plh0387i05 plh0387i07 plh0387i08 plh0387i09 plh0387i10 plh0387i11 plh0393 plh0394 plh0395i04 plh0396i01 plh0396i02 plh0396i03 plh0396i04 plh0397i05 plh0407 pli0080 pli0081 pli0082 pli0083 pli0089 plj0047 plh0195 plh0337_v2 plh0338_v2 plj0439 plb0073_h plb0036_h plh0037 plh0155 plh0188 plh0192 plh0357 plh0360 plh0366 plj0438 plb0072_h plb0568_h plc0113 plh0258_h plb0616 plc0014_h ple0179 ple0181 plh0039 plh0042 plh0129 plh0162 plh0166 plh0173 plh0183 plh0190 plh0193 plh0194 plh0196 plh0253 plh0359 plh0364 plh0365 plb0037_h plc0111 plc0112 plc0114 plb0024_h plb0041_h plb0112 plb0113 plb0114 plb0115 plb0116 plb0117 ple0178 ple0180 ple0182 plh0036 plh0179 plh0189 plh0191 plh0214 plh0254 plh0335 plh0339 plh0340 plh0341 plh0342 plh0358 plh0361 plh0362 plh0363 plh0377_v2 plj0441 plb0022_h pld0131_h pld0132_h pld0298_h plc0552 pld0047 ple0028 ple0081_h ple0177 plh0038 plh0175 plh0176 plh0184 plh0206i02 plh0206i03 plh0206i05 plh0206i10 plh0212 plh0213 plh0215 plh0216 plh0217 plh0218 plh0219 plh0220 plh0221 plh0222 plh0223 plh0224 plh0225 plh0226 plh0244 plh0255 plh0334 plh0336 plh0343_h plh0379_v2 plh0381_v2 pli0098_h plj0440 plj0442 plj0443 ple0026 ple0027 ple0029 ple0030 ple0031 ple0032 ple0033 ple0034 ple0035 ple0036 plh0007 plh0032 plh0033 plh0035 plh0040 plh0107 plh0171 plh0172 plh0174 plh0177 plh0178 plh0180 plh0182 plh0185 plh0186 plh0187 plh0204_h plh0206i01 plh0206i04 plh0206i06 plh0206i07 plh0206i08 plh0206i09 plh0206i11 plh0263_h plh0264_h plh0378_v2 plh0380_v2 plh0382_v2 plh0383_v2 plh0384_v2 plh0385_v2 plh0386_v2 pli0090_h pli0093_h pli0096_h plj0589 plb0594 ple0097_v1 plh0104 plh0105 plh0106 plh0108 plh0109 plh0110 plh0111 plh0112 plh0164 plh0344 pli0092_h pli0097_h plj0043 plj0061 plj0063 plj0104 plj0105 plj0175 plj0587 plj0588 plj0713 plh0034 pli0091_h plj0060 plj0062 plh0135 plh0134 plh0136 plh0156 pli0094_h pli0095_h plb0443 plh0199 plh0198 ple0095 plh0197 plh0202 plh0201 plg0266 {

disp "`: var label `var''" ", `var'"

}

********************************************************************************

* workers

eststo clear

estimates clear

use afd.dta, clear

label var worker_pgstib "worker"

foreach var of varlist afd_pref_vs_oth sd_sorgen_zuwanderung ///

worker_pgstib {

drop if `var'==.

}

* null

xtlogit afd_pref_vs_oth, or vce(robust)

local ll0 = e(ll)

* concern immigration

xtlogit afd_pref_vs_oth sd_sorgen_zuwanderung, or vce(robust)

estimates store m1

local ll = e(ll)

estadd scalar pr2 = (`ll0' - `ll')/`ll0'

* worker added

xtlogit afd_pref_vs_oth sd_sorgen_zuwanderung worker ///

, or vce(robust)

estimates store m3

local ll = e(ll)

estadd scalar pr2 = (`ll0' - `ll')/`ll0'

esttab * using TableOA7.rtf, replace stat(N N_clust pr2) mtitle label eform note("All effect sizes standardized to 1=1 sd") nogaps b(%9.3g) wide

shell open TableOA7.rtf

* in which year did workers switch to the AfD?

xtlogit afd_pref_vs_oth i.syear worker worker#i.syear, or vce(robust)

coefplot, base

, keep(*#*.syear)

* repeat everything for workers only

keep if worker_pgstib==1

foreach var of varlist afd_pref_vs_oth sd_sorgen_zuwanderung ///

sd_zufr_hh_eink sd_zufr_pers_eink sd_zufr_leben sd_perc_hh_net_equiv_inc sd_pgisced97 ///

sd_sorgen_eig_wirt_sit sd_sorgen_allg_wirt_sit {

drop if `var'==.

}

eststo clear

estimates clear

* null

xtlogit afd_pref_vs_oth, or vce(robust)

local ll0 = e(ll)

* concerned immigration

xtlogit afd_pref_vs_oth sd_sorgen_zuwanderung, or vce(robust)

estimates store Concern_immigration

local ll = e(ll)

estadd scalar pr2 = (`ll0' - `ll')/`ll0'

* all

xtlogit afd_pref_vs_oth sd_sorgen_zuwanderung ///

sd_pgisced97 sd_perc_hh_net_equiv_inc sd_zufr_hh_eink sd_zufr_pers_eink sd_zufr_leben ///

sd_sorgen_eig_wirt_sit sd_sorgen_allg_wirt_sit ///

, or vce(robust)

estimates store All

local ll = e(ll)

estadd scalar pr2 = (`ll0' - `ll')/`ll0'

esttab Concern_immigration Objective_deprivation Feared_deprivation All using Tablex.rtf, replace stat(N N_clust pr2) mtitle label eform note("All effect sizes standardized to 1=1 sd") nogaps b(%9.4g)

shell open Tablex.rtf

********************************************************************************

* delete old files

shell rm *.gph

shell rm *.rtf

shell rm *.csv

shell rm *.png

shell rm afd_temp.dta
