# Supplementary material for: Supporters of Germany’s far-right AfD party are concerned about immigration rather than feeling deprived
Source: PLoS One. 2026 Jul 21;21(7):e0350403. doi: 10.1371/journal.pone.0350403 (PMC13387537; doi:10.1371/journal.pone.0350403)
Supplement: S2 File — (PDF) [file pone.0350403.s002.pdf]

```
global working "/Users/martin/Documents/Stata"
cd $working
set scheme cblind1
set processor 6
label language EN

use "soepv41/pl.dta", clear
sort pid syear

merge 1:1 pid syear using "soepv41/ppathl.dta", keepusing(phrf)
drop if _merge ==2 // useless if only in ppathl
drop _merge

merge 1:1 pid syear using "soepv41/pequiv.dta", keepusing(i11102 i11110 d11106 x11104ll d11102ll d11101 l11101)
drop if _merge ==2 // useless if only in pequiv
drop _merge

recode d11102ll (2=0 "Female") (1=1 "Male") (else=.), gen("man")
generate age =d11101 if d11101>=0
clonevar state=l11101


merge 1:1 pid syear using "soepv41/pgen.dta", keepusing (pgisced97 pgstib)
drop if _merge ==2 // useless if only in pgen

gen worker_pgstib = inrange(pgstib,200,320)
tab   pgstib if worker_pgstib == 0


label language EN

label var worker_pgstib "worker based on occupational classification"
label define worker_pgstib 1 "worker" 0 "not worker"
label values worker_pgstib worker_pgstib



foreach var of varlist i11102 i11110 d11106 {
replace `var'=. if `var'<0
```

```
40 sum `var'
41 }
42
43 gen ind_inc=i11110
44
45 gen hh_net_equiv_inc=i11102/(d11106^.5)
46 xtset pid syear
47 sort pid syear
48 replace hh_net_equiv_inc=l.hh_net_equiv_inc if l.
   hh_net_equiv_inc!=. & hh_net_equiv_inc==.
49 replace hh_net_equiv_inc=l2.hh_net_equiv_inc if l2.
   hh_net_equiv_inc!=. & hh_net_equiv_inc==.
50
51 drop if syear<2013
52
53 *AfD variables
54 recode plh0012_h (27=1) (1/6 9/11 13/17 20 22/24=0) (
   else=.) , gen(afd_pref_vs_oth)
55 recode plh0012_h (27/31=1) (-4=.) (else=0) , gen(
   afd_pref_vs_all_oth)
56 recode plh0333 (1/6 9/20 22/24 = 0) (27 30 31 = 1) (
   else=.), gen(afd_vote)
57 label var afd_pref_vs_all_oth "AfD pref vs all"
58 label var afd_vote "AfD vote vs establ party"
59
60
61 * drop if ind var unavailable
62 drop if afd_pref_vs_oth==. & afd_pref_vs_all_oth==. &
   afd_vote==.
63
64 sum afd_pref_vs_oth afd_pref_vs_all_oth afd_vote
65
66 keep pid syear plh* plj* *afd* ind_inc hh_net_equiv_inc
    pgisced97 ind_inc hh_net_equiv_inc phrf i11102 i11110
   d11106 x11104ll man age worker_pgstib state // only
   keep necessary variables
67
68
69
70 foreach var of varlist plh0155 plh0171 plh0172 plh0175
   plh0176 plh0177 plh0178 plh0180 plh0182 plh0032 plh0033
    plh0035 plh0036 plh0037 plh0038 plh0040 plh0335
   plh0336 plj0046 plj0047 {
71 * sum `var' if afd_pref_vs_oth==1 // how many cases
   with AfD pref
```

```
72  di "clonevar" " " `"`: var label `var'`"' " " "=" " "
    "`var'"
73  }
74
75
76
77  clonevar zufr_hh_eink = plh0175
78  clonevar zufr_pers_eink = plh0176
79  clonevar zufr_wohnung = plh0177
80  clonevar zufr_freizeit = plh0178
81  clonevar zufr_fam = plh0180
82  clonevar zufr_leben = plh0182
83  clonevar sorgen_allg_wirt_sit = plh0032
84  clonevar sorgen_eig_wirt_sit = plh0033
85  clonevar sorgen_eig_gesund = plh0035
86  clonevar sorgen_umwelt = plh0036
87  clonevar sorgen_klima = plh0037
88  clonevar sorgen_frieden = plh0038
89  clonevar sorgen_kriminal = plh0040
90  clonevar sorgen_zuwanderung = plj0046
91  clonevar sorgen_auslaenderf = plj0047 // simply
    doubles sorgen_zuwanderung
92  clonevar     zufr_demokr=plh0152_v2
93
94
95  drop if sorgen_zuwanderung<0 // makes no sense if
    important variable not available
96
97  foreach var of varlist * {
98  capture replace `var'=. if `var'<0
99  }
100
101
102 foreach var of varlist sorgen* {
103     revrs `var', replace
104 }
105 label values sorgen_* .
106
107 egen perc_ind_inc=xtile(ind_inc), n(100) by(syear)
108 egen perc_hh_net_equiv_inc=xtile(hh_net_equiv_inc), n(
    100) by(syear)
109 replace pgisced97=. if pgisced97==0
110
111 missings dropvars *, force
112 foreach var of varlist zufr_* sorgen_* perc_ind_inc
```

```
    perc_hh_net_equiv_inc pgisced97 {
113     sum `var'
114     egen sd_`var'=std(`var')
115     sum sd_`var'
116 }
117
118 foreach var of varlist sd_* {
119 disp    "`var' " "`var' "
120 }
121
122
123 label var afd_pref_vs_oth "AfD support"
124 label var sorgen_zuwanderung "Concern immi"
125 label var zufr_hh_eink "Satisf hh inc"
126 label var zufr_pers_eink "Satisf pers inc"
127 label var zufr_leben "Satisf life"
128 label var perc_hh_net_equiv_inc "Percentile hh net
    equiv inc"
129 label var pgisced97 "Education PGISCED 97 scale"
130 label var sorgen_eig_wirt_sit "Concern own finan"
131 label var sorgen_allg_wirt_sit "Concern Ger economy"
132 label var zufr_demokr "Satisfaction democracy"
133
134
135
136 label var sd_zufr_hh_eink           "Satisf hh inc"
137 label var sd_zufr_pers_eink         "Satisf pers inc"
138 label var sd_zufr_wohnung           "Satisf flat"
139 label var sd_zufr_freizeit          "Satisf leisure"
140 label var sd_zufr_fam               "Satisf family"
141 label var sd_zufr_leben            "Satisf life"
142 label var sd_sorgen_allg_wirt_sit   "Concern econ"
143 label var sd_sorgen_eig_wirt_sit    "Concern finan"
144 label var sd_sorgen_eig_gesund      "Concern health"
145 label var sd_sorgen_umwelt          "Concern environm"
146 label var sd_sorgen_klima           "Concern climate"
147 label var sd_sorgen_frieden         "Concern peace"
148 label var sd_sorgen_kriminal        "Concern criminal"
149 label var sd_sorgen_zuwanderung     "Concern immi"
150 label var sd_sorgen_auslaenderf     "Concern xenoph"
151 label var sd_perc_hh_net_equiv_inc  "Perc hh inc"
152 label var sd_pgisced97              "Education"
153
154
155
```

```
156
157 *ssc install r2_mz
158
159 * so that all effects are positive
160 revrs sd_sorgen_auslaenderf sd_sorgen_klima
    sd_sorgen_frieden sd_zufr_hh_eink sd_zufr_wohnung
    sd_zufr_pers_eink sd_sorgen_umwelt sd_zufr_leben
    sd_perc_ind_inc sd_perc_hh_net_equiv_inc sd_pgisced97
    sd_zufr_demokr
161 label var revsd_sorgen_auslaenderf "unw xenophobia"
162 label var revsd_sorgen_klima "unw climate"
163 label var revsd_sorgen_frieden "un peace"
164 label var revsd_zufr_hh_eink "unsat hh inc "
165 label var revsd_zufr_wohnung "unsat flat"
166 label var revsd_zufr_pers_eink "unsat pers inc"
167 label var revsd_sorgen_umwelt "unw environm"
168 label var revsd_zufr_leben "unsat life"
169 label var revsd_perc_ind_inc "low pers inc"
170 label var revsd_perc_hh_net_equiv_inc "low hh net inc"
171 label var revsd_pgisced97 "low edu"
172 label var revsd_zufr_demokr "unsat democr"
173
174 format afd_pref_vs_oth sorgen_zuwanderung zufr_hh_eink
    zufr_pers_eink zufr_leben perc_hh_net_equiv_inc
    pgisced97 sorgen_eig_wirt_sit sorgen_allg_wirt_sit
    zufr_demokr %9.1f
175
176 sort pid syear
177
178 bysort pid (syear): gen last_weight = phrf[_N]
179
180
181
182 global working "/Users/martin/Documents/Stata/"
183 cd $working
184 save afd.dta, replace
185 use afd.dta, clear
186 sum syear
187 global max_year = r(max)
188 disp $max_year
189
190 ************************************************************
    ************************
191 *descriptive
192 *ssc install fsum
```

```
193 * TableOA1
194 foreach var of varlist afd_pref_vs_oth
    sd_sorgen_zuwanderung ///
195      sd_zufr_hh_eink sd_zufr_pers_eink sd_zufr_leben
    sd_perc_hh_net_equiv_inc sd_pgisced97 ///
196      sd_sorgen_eig_wirt_sit sd_sorgen_allg_wirt_sit {
197         drop if `var'==.
198      }
199
200 fsum afd_pref_vs_oth sorgen_zuwanderung zufr_hh_eink
    zufr_pers_eink zufr_leben perc_hh_net_equiv_inc
    pgisced97 sorgen_eig_wirt_sit sorgen_allg_wirt_sit,
    stat(n mean sd min max) format(%8.2f) uselabel
201
202 **********************************************************
    ************************
203 *bivariate
204 pwcorr afd_pref_vs_oth sd_sorgen_zuwanderung
    revsd_pgisced97 revsd_perc_hh_net_equiv_inc
    revsd_zufr_hh_eink revsd_zufr_pers_eink
    revsd_zufr_leben sd_sorgen_eig_wirt_sit
    sd_sorgen_allg_wirt_sit, star(.01)
205
206
207 *annually
208 estimates clear
209 eststo clear
210 use $working/afd.dta, clear
211 keep if afd_pref_vs_oth!=.
212
213
214 * afd is one-issue party in almost every year
215 foreach num of numlist 2014/2024 {
216 preserve
217 keep if syear==`num'
218 tab syear
219 eststo syear`num': logit afd_pref_vs_oth
    sd_sorgen_zuwanderung   sd_zufr_hh_eink
    sd_zufr_pers_eink sd_zufr_leben
    sd_perc_hh_net_equiv_inc sd_pgisced97
    sd_sorgen_eig_wirt_sit sd_sorgen_allg_wirt_sit [pweight
    =phrf], vce(robust)
220 margins, dydx(*) atmeans post
221 estimates store margins_`num'
222 coefplot margins_`num', rescale(100) xline(0) drop(
```

```
    mlabpos(2) title(`num') saving(syear`num'.gph, replace)
     sort labels xlabel(-2(1)5,nogrid) grid(none)
223 restore
224 }
225
226 esttab syear* using OA2.rtf, replace stat(N r2_p)
    mtitle label eform note("All effect sizes standardized
    to 1=1 sd") nogaps b(%9.3g)
227 shell open OA2.rtf
228
229 graph combine syear2014.gph syear2015.gph syear2016.gph
     syear2017.gph syear2018.gph syear2019.gph syear2020.
    gph syear2021.gph syear2022.gph syear2023.gph syear2024
    .gph, scale(1) imargin(zero) col(2) note("All effect
    sizes average marginal effects of % change AfD
    sympathy based on variables standardized to sd=1")
    ysize(8)
230 graph export Figure1.png, replace
231
232
233 sum afd_pref_vs_oth if syear==2014 [aweight=phrf]
234 sum afd_pref_vs_oth if syear==$max_year [aweight=
    last_weight]
235
236 ************************************************************
    ************************
237 cd $working/
238 use $working/afd.dta, clear
239
240 foreach var of varlist afd_pref_vs_oth
    sd_sorgen_zuwanderung ///
241      sd_zufr_hh_eink sd_zufr_pers_eink sd_zufr_leben
    sd_perc_hh_net_equiv_inc sd_pgisced97 ///
242      sd_sorgen_eig_wirt_sit sd_sorgen_allg_wirt_sit {
243         drop if `var'==.
244      }
245
246
247
248 eststo clear
249 estimates clear
250
251 * null
252 xtlogit afd_pref_vs_oth, or vce(robust)
253 local ll0 = e(ll)
```

```
* concerned immigration
xtlogit afd_pref_vs_oth sd_sorgen_zuwanderung, or vce(robust)
estimates store Concern_immigration
local ll = e(ll)
estadd scalar pr2 = (`ll0' - `ll')/`ll0'

* actual deprivation
xtlogit afd_pref_vs_oth  sd_sorgen_zuwanderung ///
     sd_pgisced97 sd_perc_hh_net_equiv_inc sd_zufr_hh_eink sd_zufr_pers_eink sd_zufr_leben ///
, or vce(robust)
estimates store Objective_deprivation
local ll = e(ll)
estadd scalar pr2 = (`ll0' - `ll')/`ll0'

* feared deprivation
xtlogit afd_pref_vs_oth sd_sorgen_zuwanderung ///
     sd_sorgen_eig_wirt_sit sd_sorgen_allg_wirt_sit ///
, or vce(robust)
estimates store Feared_deprivation
local ll = e(ll)
estadd scalar pr2 = (`ll0' - `ll')/`ll0'

* all
xtlogit afd_pref_vs_oth sd_sorgen_zuwanderung ///
     sd_pgisced97 sd_perc_hh_net_equiv_inc sd_zufr_hh_eink sd_zufr_pers_eink sd_zufr_leben ///
     sd_sorgen_eig_wirt_sit sd_sorgen_allg_wirt_sit ///
, or vce(robust)
estimates store All
local ll = e(ll)
estadd scalar pr2 = (`ll0' - `ll')/`ll0'

* last year's deprivation
xtlogit afd_pref_vs_oth sd_sorgen_zuwanderung ///
     sd_pgisced97 l.sd_perc_hh_net_equiv_inc l.sd_zufr_hh_eink l.sd_zufr_pers_eink l.sd_zufr_leben ///
     l.sd_sorgen_eig_wirt_sit l.sd_sorgen_allg_wirt_sit ///
, or vce(robust)
estimates store Lagged_deprivation
local ll = e(ll)
estadd scalar pr2 = (`ll0' - `ll')/`ll0'

```

```
* Interactions
xtlogit afd_pref_vs_oth sd_sorgen_zuwanderung ///
     sd_pgisced97 sd_perc_hh_net_equiv_inc sd_zufr_hh_eink sd_zufr_pers_eink sd_zufr_leben sd_sorgen_eig_wirt_sit sd_sorgen_allg_wirt_sit ///
     c.sd_pgisced97#c.sd_sorgen_zuwanderung c.sd_perc_hh_net_equiv_inc#c.sd_sorgen_zuwanderung c.sd_zufr_hh_eink#c.sd_sorgen_zuwanderung c.sd_zufr_pers_eink#c.sd_sorgen_zuwanderung c.sd_zufr_leben#c.sd_sorgen_zuwanderung c.sd_sorgen_eig_wirt_sit#c.sd_sorgen_zuwanderung c.sd_sorgen_allg_wirt_sit#c.sd_sorgen_zuwanderung ///
, or vce(robust)
estimates store Interactions
local ll = e(ll)
estadd scalar pr2 = (`ll0' - `ll')/`ll0'

esttab Concern_immigration Objective_deprivation Feared_deprivation All Lagged_deprivation Interactions using Table1.rtf, replace stat(N N_clust ll aic bic pr2) mtitle label eform note("All effect sizes standardized to 1=1 sd; Table shows odds ratios based on Stata xtlogit re procedure with robust standard errors") nogaps b(%9.4g)
shell open Table1.rtf

* compare Model 4 effects
estimates restore All
margins, dydx(*) post atmeans
estimates store margins
estimates restore margins
coefplot margins, mlabel mlabformat(%9.2g) mlabpos(2) sort(1:) xline(0) title() note("All effect sizes average marginal effects of % change AfD support, based on variables standardized to sd=1") saving(Figure2.gph, replace) legend(col(1)) legend(off) scale(1.3) xlabel(,nogrid) grid(none) rescale(100)
graph export Figure2.png, replace

**********************************************************************
* margins for guy who is perfect but concerned about immigration and contrary
cd $working/
use afd.dta, clear
```

```
319
320 xtlogit afd_pref_vs_oth  sorgen_zuwanderung  pgisced97
      perc_hh_net_equiv_inc  zufr_hh_eink  zufr_pers_eink
    zufr_leben sorgen_eig_wirt_sit sorgen_allg_wirt_sit ,
    or vce(robust)
321
322
323 margins, at(sorgen_zuwanderung==3  pgisced97==6
    perc_hh_net_equiv_inc==100  zufr_hh_eink=10
    zufr_pers_eink==10  zufr_leben==10 sorgen_eig_wirt_sit
    ==1 sorgen_allg_wirt_sit==1) ///
324 at(sorgen_zuwanderung==1  pgisced97==1
    perc_hh_net_equiv_inc==1  zufr_hh_eink=0
    zufr_pers_eink==0  zufr_leben==0 sorgen_eig_wirt_sit==3
     sorgen_allg_wirt_sit==3) atmeans post
325 estimates store margins
326
327 * compare indidual here...
328 coefplot margins, ylabel(1 "immigr-unconc depriv loser"
     2 "immigrat-concerned winner", angle(90) alternate)
    mlabel mlabformat(%9.1f) mlabposition(2) xtitle(
    Probability AfD-support) scale(1.3) mlabsize(small)
    xlabel(,nogrid) grid(none) ysize(1) xsize(1.5) rescale(
    100)
329 graph export Figure3.png, replace
330
331
332
333 ************************************************************
    ************************
334 * FE
335 cd $working/
336 use afd.dta, clear
337
338 foreach var of varlist afd_pref_vs_oth
    sd_sorgen_zuwanderung ///
339      sd_zufr_hh_eink sd_zufr_pers_eink sd_zufr_leben
    sd_perc_hh_net_equiv_inc  ///
340      sd_sorgen_eig_wirt_sit sd_sorgen_allg_wirt_sit {
341         drop if `var'==.
342      }
343
344
345 eststo clear
346 estimates clear
```

```
347
348
349 * concerned immigration
350 xtlogit afd_pref_vs_oth sd_sorgen_zuwanderung, or fe
    vce(boot)
351 estimates store Concern_immigration
352
353
354 * actual deprivation
355 xtlogit afd_pref_vs_oth  sd_sorgen_zuwanderung ///
356       sd_perc_hh_net_equiv_inc sd_zufr_hh_eink
    sd_zufr_pers_eink sd_zufr_leben ///
357 , or fe vce(boot)
358 estimates store Objective_deprivation
359
360
361 * feared deprivation
362 xtlogit afd_pref_vs_oth sd_sorgen_zuwanderung ///
363      sd_sorgen_eig_wirt_sit sd_sorgen_allg_wirt_sit ///
364 , or fe vce(boot)
365 estimates store Feared_deprivation
366
367
368 * all
369 xtlogit afd_pref_vs_oth sd_sorgen_zuwanderung ///
370       sd_perc_hh_net_equiv_inc sd_zufr_hh_eink
    sd_zufr_pers_eink sd_zufr_leben ///
371      sd_sorgen_eig_wirt_sit sd_sorgen_allg_wirt_sit ///
372 , or fe vce(boot)
373 estimates store All
374
375 sort pid syear
376
377 * last year's deprivation
378 xtlogit afd_pref_vs_oth sd_sorgen_zuwanderung ///
379      l.sd_perc_hh_net_equiv_inc l.sd_zufr_hh_eink l.
    sd_zufr_pers_eink l.sd_zufr_leben ///
380      l.sd_sorgen_eig_wirt_sit l.sd_sorgen_allg_wirt_sit
      ///
381 , or fe vce(boot)
382 estimates store Lagged_deprivation
383
384 * Interactions
385 xtlogit afd_pref_vs_oth sd_sorgen_zuwanderung ///
386      sd_perc_hh_net_equiv_inc sd_zufr_hh_eink
```

```
    sd_zufr_pers_eink sd_zufr_leben sd_sorgen_eig_wirt_sit
    sd_sorgen_allg_wirt_sit ///
387       c.sd_perc_hh_net_equiv_inc#c.sd_sorgen_zuwanderung
     c.sd_zufr_hh_eink#c.sd_sorgen_zuwanderung c.
    sd_zufr_pers_eink#c.sd_sorgen_zuwanderung c.
    sd_zufr_leben#c.sd_sorgen_zuwanderung c.
    sd_sorgen_eig_wirt_sit#c.sd_sorgen_zuwanderung c.
    sd_sorgen_allg_wirt_sit#c.sd_sorgen_zuwanderung ///
388 , fe or vce(boot)
389 estimates store Interactions
390
391
392 esttab Concern_immigration Objective_deprivation
    Feared_deprivation All Lagged_deprivation Interactions
    using Table3.rtf, replace stat(N N_clust ll aic bic
    r2_p) mtitle label eform note("All effect sizes
    standardized to 1=1 sd, Standard errors based on
    bootstrapping, as xtlogit fe does not allow robust
    standard errors") nogaps b(%9.3g)
393 shell open Table3.rtf
394
395
396
397 * compare Model 4 effects
398 estimates restore All
399 margins, dydx(*) post atmeans
400 estimates store margins
401 estimates restore margins
402 coefplot margins, mlabel mlabformat(%9.2g) mlabpos(2)
    sort(1:) xline(0) title() note("All effect sizes
    average marginal effects of % change AfD support,
    based on variables standardized to sd=1") saving(
    Figure4.gph, replace) legend(col(1)) legend(off) scale(
    1.3) xlabel(,nogrid) grid(none) rescale(100)
403 graph export Figure4.png, replace
404
405 ********************************************************
    ************************
406
407 *State-level AfD effect
408 cd $working/
409 use afd.dta, clear
410
411
412 foreach var of varlist afd_pref_vs_oth
```

```
    sd_sorgen_zuwanderung ///
413       sd_zufr_hh_eink sd_zufr_pers_eink sd_zufr_leben
    sd_perc_hh_net_equiv_inc sd_pgisced97 ///
414       sd_sorgen_eig_wirt_sit sd_sorgen_allg_wirt_sit {
415          drop if `var'==.
416       }
417
418 bysort state syear: egen av_sd_sorgen_zuwanderung =
    mean(sd_sorgen_zuwanderung)
419 label var av_sd_sorgen_zuwanderung "Popul concern immi"
420
421 egen cstate = group(state syear), label
422
423 *collapse av_sd_sorgen_zuwanderung afd_pref_vs_oth
    sd_sorgen_zuwanderung, by(cstate)
424 *scatter  afd_pref_vs_oth av_sd_sorgen_zuwanderung,
    mlabel(cstate) msymbol(none) scale(.5)
425
426 eststo clear
427 estimates clear
428
429 * null
430 melogit afd_pref_vs_oth || _all: R.syear || state:  ,
    or  difficult
431 local ll0 = e(ll)
432
433 * concerned immigration
434 melogit afd_pref_vs_oth sd_sorgen_zuwanderung
    av_sd_sorgen_zuwanderung || _all: R.syear || state:  ,
    or difficult
435 estimates store Concern_immigration
436 local ll = e(ll)
437 estadd scalar pr2 = (`ll0' - `ll')/`ll0'
438
439 * all
440 melogit afd_pref_vs_oth sd_sorgen_zuwanderung
    av_sd_sorgen_zuwanderung ///
441       sd_pgisced97 sd_perc_hh_net_equiv_inc
    sd_zufr_hh_eink sd_zufr_pers_eink sd_zufr_leben ///
442       sd_sorgen_eig_wirt_sit sd_sorgen_allg_wirt_sit ||
    _all: R.syear || state:  , or  difficult
443 estimates store All
444 local ll = e(ll)
445 estadd scalar pr2 = (`ll0' - `ll')/`ll0'
446
```

```
esttab Concern_immigration All using TableOA3.rtf, replace stat(N N_clust pr2) mtitle label eform note("All effect sizes standardized to 1=1 sd") nogaps b(%9.4g)
shell open TableOA3.rtf

* compare Model 4 effects
estimates restore All
margins, dydx(*) post atmeans
estimates store margins
coefplot margins, drop(_cons) mlabel mlabformat(%9.2g) mlabpos(2) sort(1:) xline(0) title() note("All effect sizes average marginal effects of % change AfD sympathy, based on variables standardized to sd=1") saving(X.gph, replace) legend(col(1)) legend(off) scale(1.3) xlabel(,nogrid) grid(none) rescale(100)
graph export FigureOA1.png, replace


**********************************************************************
* other coding of AfD
eststo clear
estimates clear
use afd.dta, clear

foreach var of varlist sd_sorgen_zuwanderung ///
     sd_zufr_hh_eink sd_zufr_pers_eink sd_zufr_leben sd_perc_hh_net_equiv_inc sd_pgisced97 ///
     sd_sorgen_eig_wirt_sit sd_sorgen_allg_wirt_sit {
        drop if `var'==.
     }

foreach var of varlist afd_pref_vs_all_oth afd_vote {
preserve
keep if `var'!=.
* null
xtlogit `var', or vce(robust)
local ll0 = e(ll)

* concerned immigration
xtlogit `var' sd_sorgen_zuwanderung, or vce(robust)
estimates store `var'
local ll = e(ll)
estadd scalar pr2 = (`ll0' - `ll')/`ll0'
```

```
482
483 * all
484 xtlogit `var' sd_sorgen_zuwanderung  ///
485      sd_pgisced97 sd_perc_hh_net_equiv_inc
    sd_zufr_hh_eink sd_zufr_pers_eink sd_zufr_leben ///
486      sd_sorgen_eig_wirt_sit sd_sorgen_allg_wirt_sit ///
487 , or vce(robust)
488 estimates store `var'_depriv
489 local ll = e(ll)
490 estadd scalar pr2 = (`ll0' - `ll')/`ll0'
491 restore
492 }
493
494 esttab * using TableOA4.rtf, replace stat(N N_clust pr2
    ) mtitle label eform note("All effect sizes
    standardized to 1=1 sd") nogaps b(%9.3g)
495 shell open TableOA4.rtf
496
497 * compare effects of immigration-concerns across models
498 coefplot *, keep(sd_sorgen_zuwanderung) mlabel
    mlabformat(%3.2g) mlabpos(2) eform title() note("All
    effect sizes standardized") saving(btw.gph, replace)
    legend(col(1)) scale(1.2) xlabel(1(1)10) plotlabels(
    "before controls" "after adjusting for deprivation"
    "after adjusting for feared deprivation" "after
    adjusting for all types of deprivation") ylabel(1
    "Effect of immigration-concern on AfD-support", angle(
    90))
499
500
501
502
503
504 ************************************************************
    ************************
505 * adjusting for demographics
506 * other coding of AfD
507 eststo clear
508 estimates clear
509 use afd.dta, clear
510
511 foreach var of varlist afd_pref_vs_oth
    sd_sorgen_zuwanderung ///
512      sd_zufr_hh_eink sd_zufr_pers_eink sd_zufr_leben
    sd_perc_hh_net_equiv_inc sd_pgisced97 ///
```

```
513       sd_sorgen_eig_wirt_sit sd_sorgen_allg_wirt_sit man
     age state {
514          drop if `var'==.
515       }
516
517   * null
518   xtlogit afd_pref_vs_oth, or vce(robust)
519   local ll0 = e(ll)
520
521   * concerned immigration
522   xtlogit afd_pref_vs_oth sd_sorgen_zuwanderung man c.age
      ##c.age i.state, or vce(robust)
523   estimates store m1
524   local ll = e(ll)
525   estadd scalar pr2 = (`ll0' - `ll')/`ll0'
526
527   * all
528   xtlogit afd_pref_vs_oth sd_sorgen_zuwanderung man c.age
      ##c.age i.state ///
529       sd_pgisced97 sd_perc_hh_net_equiv_inc
      sd_zufr_hh_eink sd_zufr_pers_eink sd_zufr_leben ///
530       sd_sorgen_eig_wirt_sit sd_sorgen_allg_wirt_sit ///
531   , or vce(robust)
532   estimates store m2
533   local ll = e(ll)
534   estadd scalar pr2 = (`ll0' - `ll')/`ll0'
535
536   esttab * using TableOA5.rtf, replace stat(N N_clust pr2
      ) mtitle label eform note("All effect sizes
      standardized to 1=1 sd") nogaps b(%9.3g) wide
537   shell open TableOA5.rtf
538
539
540   ********************************************************
      ************************
541   * More variables for Online Annex
542   use afd.dta, clear
543   merge 1:1 pid syear using "soepv41/pl.dta", keepusing(
      pl*)
544
545   foreach var of varlist afd_pref_vs_oth
      sd_sorgen_zuwanderung ///
546       revsd_zufr_hh_eink revsd_zufr_pers_eink
      revsd_zufr_leben revsd_perc_hh_net_equiv_inc
      revsd_pgisced97 ///
```

```
547       sd_sorgen_eig_wirt_sit sd_sorgen_allg_wirt_sit {
548          drop if `var'==.
549       }
550
551  * Loop over the variables to check for the percentage
     of missing values
552  foreach var of varlist pl* {
553   * Calculate the percentage of missing values
554   capture replace `var'=. if `var'<0
555   qui count if missing(`var')
556   local missings = r(N)
557   local total = _N
558   local pct_missing = 100 * `missings' / `total'
559
560   * Drop the variable if the percentage exceeds the
     threshold
561   if `pct_missing' > 90 {
562   drop `var'
563   di "Dropped variable: `var' with `pct_missing'%
     missing values"
564   }
565  }
566
567  save afd_temp.dta, replace
568  use afd_temp.dta, clear
569
570
571
572  eststo clear
573  estimates clear
574
575  global cat_vars "plb0022_h plb0037_h plb0072_h
     plb0073_h  plb0568_h plc0111 plc0112 plc0113 plc0114
     pld0131_h pld0132_h pld0298_h plh0258_h"
576
577  global metric_vars "plb0024_h plb0036_h plb0041_h
     plb0112 plb0113 plb0114 plb0115 plb0116 plb0117
     plb0443 plb0594 plb0616 plc0014_h plc0552 pld0047
     ple0026 ple0027 ple0028 ple0029 ple0030 ple0031
     ple0032 ple0033 ple0034 ple0035 ple0036 ple0081_h
     ple0095 ple0097_v1 ple0177 ple0178 ple0179 ple0180
     ple0181 ple0182 ple0200 plg0266 plh0004 plh0007
     plh0032 plh0033 plh0034 plh0035 plh0036 plh0037
     plh0038 plh0039 plh0040 plh0042 plh0104 plh0105
     plh0106 plh0107 plh0108 plh0109 plh0110 plh0111
```

```
    plh0112 plh0129 plh0134 plh0135 plh0136 plh0155
    plh0156 plh0162 plh0164 plh0166 plh0171 plh0172
    plh0173 plh0174 plh0175 plh0176 plh0177 plh0178
    plh0179 plh0180  plh0182 plh0183 plh0184 plh0185
    plh0186 plh0187 plh0188 plh0189 plh0190 plh0191
    plh0192 plh0193 plh0194 plh0195 plh0196 plh0197
    plh0198 plh0199  plh0201 plh0202 plh0204_h plh0206i01
    plh0206i02 plh0206i03 plh0206i04 plh0206i05 plh0206i06
    plh0206i07 plh0206i08 plh0206i09 plh0206i10 plh0206i11
    plh0212 plh0213 plh0214 plh0215 plh0216 plh0217
    plh0218 plh0219 plh0220 plh0221 plh0222 plh0223
    plh0224 plh0225 plh0226 plh0244 plh0253 plh0254
    plh0255 plh0263_h plh0264_h plh0334 plh0335 plh0336
    plh0337_v2 plh0338_v2 plh0339 plh0340 plh0341 plh0342
    plh0343_h plh0344     plh0357 plh0358 plh0359 plh0360
    plh0361 plh0362 plh0363 plh0364 plh0365 plh0366
    plh0377_v2 plh0378_v2 plh0379_v2 plh0380_v2 plh0381_v2
    plh0382_v2 plh0383_v2 plh0384_v2 plh0385_v2 plh0386_v2
    plh0387i01 plh0387i02 plh0387i03 plh0387i04 plh0387i05
    plh0387i06 plh0387i07 plh0387i08 plh0387i09 plh0387i10
    plh0387i11 plh0390 plh0391 plh0392 plh0393 plh0394
    plh0395i01 plh0395i02 plh0395i03 plh0395i04 plh0395i05
    plh0395i06 plh0396i01 plh0396i02 plh0396i03 plh0396i04
    plh0397i01 plh0397i02 plh0397i03 plh0397i04 plh0397i05
    plh0407 plh0408 plh0409 plh0410 pli0079 pli0080
    pli0081 pli0082 pli0083 pli0089 pli0090_h pli0091_h
    pli0092_h pli0093_h pli0094_h pli0095_h pli0096_h
    pli0097_h pli0098_h plj0047 plj0043 plj0060 plj0061
    plj0062 plj0063 plj0104 plj0105 plj0175 plj0433_h
    plj0434_h plj0435_h plj0436_h plj0437_h plj0438
    plj0439 plj0440 plj0441 plj0442 plj0443 plj0587
    plj0588 plj0589 plj0713 plj0723 plj0724"
578
579
580 * categorical vars
581 foreach var of varlist $cat_vars {
582 local label: variable label `var' // remove commas
    from labels
583 local newlabel = subinstr("`label'", ",", "", .)
584 label variable `var' "`newlabel'"
585 egen std_`var'=std(`var')
586 logit afd_pref_vs_oth sd_sorgen_zuwanderung i.`var', or
     vce(robust)
587 estimates store `var'
588 }
```

```
592 foreach var of varlist $metric_vars {
593 local label: variable label `var' // remove commas
    from labels
594 local newlabel = subinstr("`label'", ",", "", .)
595 label variable `var' "`newlabel'"
596 egen std_`var'=std(`var')
597 label var std_`var' "`: var label `var'' SD"
598 logit afd_pref_vs_oth sd_sorgen_zuwanderung std_`var',
    or vce(robust)
599 estimates store `var'
600 }
601
602 * Use Excel for Online Annex
603 esttab * using other_vars.csv, replace stat(N r2_p)
    mtitle label eform note("All effect sizes standardized
    to 1=1 sd") nogaps b(%9.3g) compress not
604 shell open -a "Microsoft Excel" "other_vars.csv"
605
606
607 foreach var of varlist plj0435_h ple0200 plj0434_h
    plj0724 plj0433_h plj0437_h plh0395i01 plj0436_h
    plh0387i06 plh0392 plh0395i02 plh0395i05 plh0395i06
    plh0397i01 plh0408 plh0409 plh0410 plh0390 plh0391
    plh0395i03 plh0397i02 plh0397i03 plh0397i04 pli0079
    plj0723 plh0004 plh0387i01 plh0387i02 plh0387i03
    plh0387i04 plh0387i05 plh0387i07 plh0387i08 plh0387i09
    plh0387i10 plh0387i11 plh0393 plh0394 plh0395i04
    plh0396i01 plh0396i02 plh0396i03 plh0396i04 plh0397i05
    plh0407 pli0080 pli0081 pli0082 pli0083 pli0089 plj0047
     plh0195 plh0337_v2 plh0338_v2 plj0439 plb0073_h
    plb0036_h plh0037 plh0155 plh0188 plh0192 plh0357
    plh0360 plh0366 plj0438 plb0072_h plb0568_h plc0113
    plh0258_h plb0616 plc0014_h ple0179 ple0181 plh0039
    plh0042 plh0129 plh0162 plh0166 plh0173 plh0183 plh0190
     plh0193 plh0194 plh0196 plh0253 plh0359 plh0364
    plh0365 plb0037_h plc0111 plc0112 plc0114 plb0024_h
    plb0041_h plb0112 plb0113 plb0114 plb0115 plb0116
    plb0117 ple0178 ple0180 ple0182 plh0036 plh0179 plh0189
     plh0191 plh0214 plh0254 plh0335 plh0339 plh0340
    plh0341 plh0342 plh0358 plh0361 plh0362 plh0363
    plh0377_v2 plj0441 plb0022_h pld0131_h pld0132_h
    pld0298_h plc0552 pld0047 ple0028 ple0081_h ple0177
    plh0038 plh0175 plh0176 plh0184 plh0206i02 plh0206i03
    plh0206i05 plh0206i10 plh0212 plh0213 plh0215 plh0216
    plh0217 plh0218 plh0219 plh0220 plh0221 plh0222 plh0223
```

```
 plh0224 plh0225 plh0226 plh0244 plh0255 plh0334
plh0336 plh0343_h plh0379_v2 plh0381_v2 pli0098_h
plj0440 plj0442 plj0443 ple0026 ple0027 ple0029 ple0030
 ple0031 ple0032 ple0033 ple0034 ple0035 ple0036
plh0007 plh0032 plh0033 plh0035 plh0040 plh0107 plh0171
 plh0172 plh0174 plh0177 plh0178 plh0180 plh0182
plh0185 plh0186 plh0187 plh0204_h plh0206i01 plh0206i04
 plh0206i06 plh0206i07 plh0206i08 plh0206i09 plh0206i11
 plh0263_h plh0264_h plh0378_v2 plh0380_v2 plh0382_v2
plh0383_v2 plh0384_v2 plh0385_v2 plh0386_v2 pli0090_h
pli0093_h pli0096_h plj0589 plb0594 ple0097_v1 plh0104
plh0105 plh0106 plh0108 plh0109 plh0110 plh0111 plh0112
 plh0164 plh0344 pli0092_h pli0097_h plj0043 plj0061
plj0063 plj0104 plj0105 plj0175 plj0587 plj0588 plj0713
 plh0034 pli0091_h plj0060 plj0062 plh0135  plh0134
plh0136 plh0156     pli0094_h pli0095_h plb0443
plh0199 plh0198 ple0095 plh0197 plh0202 plh0201 plg0266
  {
    disp "`: var label `var'" ", `var'"
}



**********************************************************
************************
* workers

eststo clear
estimates clear
use afd.dta, clear
label var worker_pgstib "worker"

foreach var of varlist afd_pref_vs_oth
sd_sorgen_zuwanderung ///
    worker_pgstib {
        drop if `var'==.
     }


* null
xtlogit afd_pref_vs_oth, or vce(robust)
local ll0 = e(ll)

* concern immigration
xtlogit afd_pref_vs_oth sd_sorgen_zuwanderung, or vce(
```

```
local ll = e(ll)
estadd scalar pr2 = (`ll0' - `ll')/`ll0'

* worker added
xtlogit afd_pref_vs_oth sd_sorgen_zuwanderung worker ///
, or vce(robust)
estimates store m3
local ll = e(ll)
estadd scalar pr2 = (`ll0' - `ll')/`ll0'

esttab * using TableOA7.rtf, replace stat(N N_clust pr2
) mtitle label eform note("All effect sizes
standardized to 1=1 sd") nogaps b(%9.3g) wide
shell open TableOA7.rtf

* in which year did workers switch to the AfD?
xtlogit afd_pref_vs_oth i.syear worker worker#i.syear,
or vce(robust)
coefplot, base
, keep(*#*.syear)



* repeat everything for workers only
keep if worker_pgstib==1

foreach var of varlist afd_pref_vs_oth
sd_sorgen_zuwanderung ///
     sd_zufr_hh_eink sd_zufr_pers_eink sd_zufr_leben
sd_perc_hh_net_equiv_inc sd_pgisced97 ///
     sd_sorgen_eig_wirt_sit sd_sorgen_allg_wirt_sit {
        drop if `var'==.
     }

eststo clear
estimates clear

* null
xtlogit afd_pref_vs_oth, or vce(robust)
local ll0 = e(ll)

* concerned immigration
xtlogit afd_pref_vs_oth sd_sorgen_zuwanderung, or vce(
robust)
estimates store Concern_immigration
```

```
673 local ll = e(ll)
674 estadd scalar pr2 = (`ll0' - `ll')/`ll0'
675
676 * all
677 xtlogit afd_pref_vs_oth sd_sorgen_zuwanderung ///
678      sd_pgisced97 sd_perc_hh_net_equiv_inc
    sd_zufr_hh_eink sd_zufr_pers_eink sd_zufr_leben ///
679      sd_sorgen_eig_wirt_sit sd_sorgen_allg_wirt_sit ///
680 , or vce(robust)
681 estimates store All
682 local ll = e(ll)
683 estadd scalar pr2 = (`ll0' - `ll')/`ll0'
684
685 esttab Concern_immigration Objective_deprivation
    Feared_deprivation All using Tablex.rtf, replace stat(N
     N_clust pr2) mtitle label eform note("All effect
    sizes standardized to 1=1 sd") nogaps b(%9.4g)
686 shell open Tablex.rtf
687
688
689
690
691 ************************************************************
    ************************
692 * delete old files
693 shell rm *.gph
694 shell rm *.rtf
695 shell rm *.csv
696 shell rm *.png
697 shell rm afd_temp.dta
698
```
