# Supplementary material for: Supporters of Germany’s far-right AfD party are concerned about immigration rather than feeling deprived
Source: PLoS One. 2026 Jul 21;21(7):e0350403. doi: 10.1371/journal.pone.0350403 (PMC13387537; doi:10.1371/journal.pone.0350403)
Supplement: Attachment — Submitted filename: R&R document.docx [file pone.0350403.s004.docx]

# R&R Report for PONE-D-25-58118: Supporters of Germany’s far-right AfD party are concerned about immigration rather than feeling deprived

**Editor’s request**: When submitting your revision, we need you to address these additional requirements. 1. Please ensure that your manuscript meets PLOS ONE's style requirements, including those for file naming. The PLOS ONE style templates can be found at

<https://journals.plos.org/plosone/s/file?id=wjVg/PLOSOne_formatting_sample_main_body.pdf> <https://journals.plos.org/plosone/s/file?id=ba62/PLOSOne_formatting_sample_title_authors_affiliations.pdf>

**Author’s response**: We went through every point of each of these documents and formatted the paper accordingly.

**Editor’s request**: 2. We are unable to open your Supporting Information file [File Name]. Please kindly revise as necessary and re-upload.

**Author’s response**: The file we uploaded was a .do file, which needs Stata to be opened. We have converted this into a pdf and uploaded it under the category of “Supporting Information” With the commands in this file, everyone who has access to the German Socio-Economic Panel, for which we have provided the addresses, can replicate our entire analysis.

**Editor’s request**: 3. We noted in your submission details that a portion of your manuscript may have been presented or published elsewhere. “We have uploaded a previous version of this article on a preprint server: <https://www.ssoar.info/ssoar/handle/document/105248> The authors that were still part of the project at the time we have uploaded this have agreed to leave the project though." Please clarify whether this [conference proceeding or publication] was peer-reviewed and formally published. If this work was previously peer-reviewed and published, in the cover letter please provide the reason that this work does not constitute dual publication and should be included in the current manuscript.

**Author’s response**: We have taken this file offline to avoid any dual publication issues. The authors who were part of the article in the Open Repository helped me with the R&R, so I decided they should be part of the project again. I have therefore added them as co-authors.

**Editor’s request**: 4. Please note that your Data Availability Statement is currently can’t access to a direct link. If your manuscript is accepted for publication, you will be asked to provide these details on a very short timeline. We therefore suggest that you provide this information now, though we will not hold up the peer review process if you are unable.

**Author’s response**: In the data availability statement, we now state that: All data is from the German Socio-Economic Panel, which can be obtained here: <https://www.leben-in-deutschland.de/?lang=en> Specifically, the SOEP v40 that we have used can be found here: <https://www.diw.de/sixcms/detail.php?id=951563> with the DOI info: 10.5684/soep.core.v40eu All replications of all results are possible with the commands that are found in the uploaded pdf that contains all our Stata commands. Thus, both the data, as well as the commands to reproduce our analysis are accessible.

**Editor’s request**: 5. If the reviewer comments include a recommendation to cite specific previously published works, please review and evaluate these publications to determine whether they are relevant and should be cited. There is no requirement to cite these works unless the editor has indicated otherwise.

**Author’s response**: We have cited the publication that the reviewer has referred to.

**Editor’s request**: I am making a decision based on a single report, in order to avoid any further delays. I suggest you carefully follow the remarks of the referee. The point about panel data would really make a difference.

**Author’s response**: We were able to incorporate all suggestions as detailed below. Particularly, we make sure to highlight our use of the SOEP as a panel and did the extra analysis that the reviewer suggest.

**Reviewer 1 request**: This is a nice empirical contribution to the literature on determinants of far right-wing party support in general and on support for the far-right Alternative for Germany in particular. The paper therefore provides new insights into a societally highly relevant and scientifically highly important topic. The authors make use of the GSOEP dataset which covers a large number of cases for every year between 2014 and 2023, which allows the authors to estimate if feeling deprived or concerns about immigration matter for considering choosing the AfD in an election. The authors, using sophisticated statistical models, show – convincingly – that concerns about immigration are driving the AfD vote, and not concerns feeling deprived. I like the paper a lot and think that the authors make an important contribution. However, there are a few aspects that the authors should consider when revising the paper. First, the paper would benefit from a clear and distinct theory section. The current version of the manuscript provides a longer introduction in which the authors present a nicely literature review. On that basis, the authors present three hypotheses. From my perspective the paper would gain significantly in its scientific impact when the authors would present a solidly derived mechanism that shows why concerns about immigration should affect AfD support more than feeling deprived. The authors could refer here to discussions in their literature review and – in particular – on changes in the AfD programmatic profile (the AfD started as a party focusing on Euroscepticism, before concentrating on migration and immigration since 2015).

**Author’s response**: Yes, we agree that our main argument, that concerns about immigration should affect AfD support more than feeling deprived, should have been given a more solidly derived mechanism. We now provide this in the introduction, right before mentioning the first hypothesis, which the reviewer rightly asks us to justify more than we did in the previous version of the paper. We therefore now argue, that, first, the AfD’s program would have little to offer to the “losers of modernization” [1], second, that the AfD actively downplays distributional conflicts in favor of cultural ones [2] and third, we now use Moral Foundations Theory in the introduction to argue that individuals on the right of the political spectrum subscribe more to a view of morality that distinguishes ingroups and outgroups [3-6]. This could make concern about immigration if not the only, then possibly the main reason why individuals feel drawn to the AfD and, as mentioned by the reviewer, it would mean that the more the AfD stokes concern about immigration, the more individuals should be drawn to it. You can see how we now argue this before Hypothesis 1, giving an explicitly argued mechanism why concerns about immigration should affect AfD support more than feeling deprived.

**Reviewer 1 request**: Secondly, I do not really understand why the authors do not make more use of the panel structure of the data the use. The main advantage of the GSOEP dataset is that it is a panel dataset and that it thus allows for drawing causal inferences, as the authors do in section 3.3 of their paper. However, the authors use the data to a large extent in their analysis as a simple survey and do not use the time-series character of the data adequately. For instance, the authors could argue in the theory section that changes in an individual’s position or attitude on immigration between the current and the previous should affect the probability to vote AfD. For example, if an individual becomes less in favour of immigration during the last year, she should be more likely to vote or support AfD.

**Author’s response**: As the reviewer suggests, we already do use the panel structure in the way that he proposes in section 3.3 of the paper. We thus indeed test whether changes in the life of a person lead to increased support for the AfD in the life of the same person. However, he is right to argue that we have not made this prominent enough in the introduction. Exactly as the reviewer suggests, we therefore now mention explicitly that we test how in an individual’s attitude on immigration between waves affects the probability to vote AfD, which was indeed not prominent enough so far.

**Reviewer 1 request**: Third, the online appendix provides several robustness checks in which the authors show that the results remain stable when controlling for selected demographic variables like net household income, sex, education, regional background, etc. I think that the authors could do a better job in not only connecting the selected control variables to standard theories of voting behaviour but could also include further variables that are explicitly relevant for analysing the support for radical right parties. For instance, the GSOEP data allows for coding the professional background of parties, and on that basis the authors could connect their study to the ongoing debate when and under which conditions workers increasingly switched to the AfD and if this social group did so in particular because of concerns about immigration. In this context, it would be extremely interesting to see when this pattern became observable during the time period under study (a recent analysis (<https://link.springer.com/article/10.1007/s11615-025-00604-y> ) using GSOEP data shows that workers, even those with a former SPD identification, switched to the AfD in 2017, but were no AfD supporters in 2013 when the AfD highlighted their Eurosceptic positions and did less focus on immigration). Along these lines, the authors could also consider doing the analysis on the basis of the pooled GSEP dataset covering all years from 2014 to 2023. On that basis, the authors could interact their substantive explanatory variables with dummy variables for each year to identify if the effects of the explanatory variables changed during the observation period.

**Author’s response**: There are a number of suggestions in this, all of which we have taken up. First, the reviewer argues that we should justify our control variables more explicitly. We have done this, now citing literature to highlight why we use each specific demographic control, and thus showing for each how the literature has linked it to AfD-support in the past.
Second, the reviewer asks to show when and under which conditions workers increasingly switched to the AfD, and how this is related to concern about immigration. This, again, is a great idea, so we have used the variable pgstib from SOEP’s dataset pgen to code the following occupational categories as workers: [200] Worker, no further information; [210] Untrained Worker; [220] Semi-Trained Worker; [230] Trained Worker; [240] Foreman, group leader; [250] Foreman; [310] Blue-collar worker in agriculture; [320] Skilled worker in agriculture We then tested whether being a worker explains AfD support better than knowing whether someone is concerned about immigration. The results show that the total explained variation of AfD support increases from r2= .0822 to r2=.085 after additionally knowing whether some is a worker, beyond already knowing whether someone is concerned about immigration. Thus, knowing whether someone is a worker only increases the total explained AfD support by 085/.0822 = 0.034 = about 3 percent. We have added this as an additional test in the robustness test section.
Third, the reviewer asks us to show whether “workers increasingly switched to the AfD […] in particular because of concerns about immigration”. We have therefore calculated a separate regression for workers, to show whether they particularly switched towards AfD support due to concern about immigration. If workers are one standard deviation more concerned about immigration, then their chance to favor the AfD increases 7.84-fold, which is very similar to our main effect of 7.58. So workers are not particularly attracted to the AfD due to concerns about immigration.
Fourth, as suggested, we also showed when specifically workers switched to the AfD by using the pooled SOEP. This indeed shows that workers were increasingly drawn to the AfD over time. But so was everyone else, as the AfD became more accepted from 2014 to 2024. Workers do not diverge from this general trend. We therefore do not contradict the result from the quoted paper, namely that workers increasingly support to AfD over time. What we do not find however, is that this trend among workers diverges from the general trend of increasing AfD support over time.

Thus, as best as we could tell, we were able to include all suggestions and thank the reviewer for these, which, in our view, improved the paper greatly.

1.	Lengfeld H. Die „Alternative für Deutschland“: eine Partei für Modernisierungsverlierer? KZfSS Kölner Zeitschrift für Soziologie und Sozialpsychologie. 2017;69(2):209–32. doi: 10.1007/s11577-017-0446-1.

2.	Rippl S, Seipel C. Modernisierungsverlierer, Cultural Backlash, Postdemokratie. KZfSS Kölner Zeitschrift für Soziologie und Sozialpsychologie. 2018;70(2):237–54. doi: 10.1007/s11577-018-0522-1.

3.	Graham J, Haidt J, Nosek BA. Liberals and conservatives rely on different sets of moral foundations. J Pers Soc Psychol. 2009;96(5):1029–46. doi: 10.1037/a0015141. PubMed PMID: 19379034.

4.	Horowitz M, Haynor A, Kickham K. Sociology’s Sacred Victims and the Politics of Knowledge: Moral Foundations Theory and Disciplinary Controversies. The American Sociologist. 2018;49(4):459–95. doi: 10.1007/s12108-018-9381-5.

5.	Franks AS, Scherr KC. Using Moral Foundations to Predict Voting Behavior: Regression Models from the 2012 U.S. Presidential Election. Analyses of Social Issues and Public Policy. 2015;15(1):213–32. doi: <https://doi.org/10.1111/asap.12074>.

6.	Graham J, Haidt J, Koleva S, Motyl M, Iyer R, Wojcik SP, et al. Moral Foundations Theory. In: Devine P, Plant A, editors. Advances in Experimental Social Psychology2013. p. 55–130.
