# Supplementary material for: Supporters of Germany’s far-right AfD party are concerned about immigration rather than feeling deprived
Source: PLoS One. 2026 Jul 21;21(7):e0350403. doi: 10.1371/journal.pone.0350403 (PMC13387537; doi:10.1371/journal.pone.0350403)
Supplement: Attachment — Submitted filename: R&R_document_auresp_2.docx [file pone.0350403.s005.docx]

**Revisions for “Supporters of Germany’s far-right AfD party are concerned about immigration rather than feeling deprived”**

**Editor’s request**: The abstract can be shortened; I would suggest to cancel “We show how an immigration-unconcerned but deprived “loser of modernization” still only has half the likelihood to support the AfD than an entirely non deprived “winner of modernization” who is concerned about immigration.” (And I would suppress “bivariate” which is not clear for a large audience; in place, I would suggest to use the term “direct” – for indicating another idea – see my point below).
**Author’s response**: We have removed the sentence in question and changed “bivariate” to “direct” throughout the paper.

**Editor’s request**: I agree with you that, statistically, immigration concern is a better (direct) predictor of the AFD vote than economic insecurity. This is your main result and your point deserves to be published. However, some elements need to be acknowledged.
1/ You did not check whether economic insecurity was (previously) the underlying cause of immigration concern. To do this, structural modelling (multiple equation estimation) would be necessary. I'm not explicitly asking for it, but the lack of a genuine analysis of opinion formation on immigration must be acknowledged.
**Author’s response**: What you are asking for is actually feasible and it is a great idea! You are suggesting that prior (rather than current) deprivation is what really explains fear of immigration, which then explains AfD support, making deprivation an antecedent to immigration concern and thus immigration concern only a proximate cause (mediator) for AfD support. Using the data’s panel structure, this can actually be tested – within limits, as one can test whether last year’s feeling of deprivation, which is causally prior to this year’s fear of immigration, diminishes the direct link between this year’s fear of immigration and this year’s AfD support. Yet prior deprivation also does not work very well in either explaining later AfD support in addition to what current fear of immigration explains, and it also does not explain away a large part of the direct link between later AfD support and later concern about immigration. This suggest that fear of immigration is not itself an influence that is due to prior deprivation. We should have tested this in prior iterations of the paper and I am therefore very glad that you have encouraged us to do so.

**Editor’s request**: 2/ I'm almost certain that, if you test, an interaction effect between immigration and deprivation (statistical effect of ‘immigration x deprivation’) on AFD-support, it would be significant. As an economist, I would be interested to see the result; this is another way to test that the formation of immigration concern and economic deprivation synergize (…or not! if I am wrong).
**Author’s response**: This is an amazing idea that yielded amazing results! First, what have we done? We have interacted each deprivation variable with concern for immigration. Then we have compared this to the main model, which shows the following: 1) “Simple” (non-interacted) concern for immigration remains as significant and as strong a predictor as in the main model, meaning that the direct effect of immigration-concern on AfD support exists irrespective of the effect that immigration-concern may have within specifically deprived groups. Also, 2) the only significant interaction effects are between immigration-concern and education as well as perceived household income, and, amazingly, these are positive, not negative, meaning that – counterintuitively, it is especially the highly educated and those who perceive themselves to have a high income who support the AfD when they fear immigration, which is the opposite of what a deprivation-centered view would predict. It is thus not being deprived that makes your fear of immigration increase your AfD support, but precisely the opposite. In addition, all other interaction effects are insignificant. In this sense, deprivation does not explain how concern about immigration leads to AfD support and if it does, it is the well-off, not the deprived, whose fear of immigration leads to AfD support. This is now Model 6 in Table 1. What you suggested are ways to test not only whether deprivation-variables explain more AfD-support than concern about immigration explains, but whether concern about immigration explains AfD support due to deprivation. Since this is an important distinction, and since you have made us introduce two additional ways to test whether the relationship between concern about immigration and AfD support could be due to deprivation, we have introduced a hypothesis 1b, which is centered on whether the link between x and y is due to z, while Hypothesis 1a tests whether x and z explain more of y than x can explain alone.

**Editor’s request**: 3/ Please reread the article. I think you're overestimating the result concerning deprivation (you can't claim that there's no effect of deprivation, or even a weak one (fixed effects model, model 2, Table 2: the effect is 1.32, p < 0.001, which is significant, even if it is smaller than that of the "cultural" variable)). Please correct the paper when you overstate the no-relationship.
**Author’s response**: True, we now made sure we speak about a “weaker” effect. Indeed, we can be much more precise. Of the variation in AfD support that we can explain, 75 percent is explained by concern about immigration and the remaining 25 percent by variables that measure deprivation. In terms of voting behavior, of all variation that we can explain, concern about immigration explains 90 percent and deprivation-variables 10 percent. We now mention these numbers right away, so that it is clear what we mean when we speak of a comparatively stronger or weaker effect.

**Editor’s request**: The panel data analysis (fixed-effect) is crucial for your demonstration. However: -Model 1 / 2 / 3 / 4 should be presented better (please, think to a table that give a recap’ on the assumptions for the four models). Then, could you have a large Table of results (estimated coefficients) with all the 4 models?
**Author’s response**: We have done exactly this.

**Editor’s request**: I don’t understand why you need bootstrapping to generate sd? (is it the same in Table 1?)
**Author’s response**: The reason is simply that fixed effects logistics models (different from random effects logistics models) do not allow for robust or person-centered standard errors, so bootstrapping is the only method to get conservative standard errors with individually-fixed logistic models for panel data. This is now mentioned in the paper.

**Editor’s request**: -Comparisons between models can be tested formally (LR test); this is better than only considering models’ respective R2.
**Author’s response**: This was what we tried before comparing r2 values. However, lr tests refuse to work with robust standard errors, as they derive their validity from the assumption that a model is correctly specified, while the Huber/White robust standard errors are needed precisely to make standard errors robust to misspecifications such as heteroskedasticity or within-person clustering, which we know exist in panel data. We can however use a Wald test to see whether all deprivation variables are jointly significant compared to a model that only contains the fear of deprivation variable. This is possible and – to be sure – they are jointly significant. But since many of them are already significant on their own, this can already be inferred from the table as it is. What we therefore do to make such model comparisons possible is to not only show the ll values of each model directly, but also the AIC and BIC values. Comparing those can show readers directly whether the more complicated models explain more variance, which they do. However, only the r2 values show just how much variance simpler models explain versus the more complicated ones. So we simply keep both the r2 values, as well as having added the ll, aic and bic, so that interested researchers can compare model fit.

**Editor’s request**: -This is very important in the panel data analysis to be more precise on the waves; What are the dates for the past values of immigration-concern and deprivation?
**Author’s response**: As indicated in the descriptives in the Online Annex (Table OA1), we have data from every single year from 2014-2024. Table OA2 also shows how much complete individual data we have for each year and the regression tables now show how many individuals we have sampled over how individual observation points. We have also mentioned in the paper that we have 11 waves, as we have data from every single wave from 2014-2024. So as far as we can tell, we were able to incorporate all your suggestions, and hope that you are satisfied with the way we did so.
