# Supplementary material for: Supporters of Germany’s far-right AfD party are concerned about immigration rather than feeling deprived
Source: PLoS One. 2026 Jul 21;21(7):e0350403. doi: 10.1371/journal.pone.0350403 (PMC13387537; doi:10.1371/journal.pone.0350403)
Supplement: Attachment — Submitted filename: R&R_document_auresp_3.docx [file pone.0350403.s006.docx]

**R&R report for :**

**Supporters of Germany’s far-right AfD party are concerned about immigration rather than feeling deprived**

**Editor’s request**: 1/ I am surprized that the study of the interaction effect is not made within the fixed-effect modelling (both variables are time-varying, this should be possible);

**Author’s response**: The reason I have not done this is that there are not enough statistical cases to perform this analysis meaningfully. For the random effects regressions, we have 31985 individuals, for which we know that they do or do not support the AfD. In contrast, for the fixed effects model, we only have 936 individuals (and thus only less than 3 % of the cases) for which we know that they have *switched to or away from the AfD over time*. This is likely not enough to not only use main effects, but to additionally interact these main effect with 6 variables. However, I have done the requested fixed effects analysis anyways now and it is in the paper. Predictably, however, every single interaction effect is insignificant, which can be due to the aforementioned low sample size or because it is a substantive non-result. In any case, it merely reiterates the main point of the paper, so it cannot hurt to do it.

**Editor’s request**: 2/ this sentence would deserve a bibliographic references, for each of the (two) propositions: "Germany might be special, as the AfD emerged as a party of economically liberal and relatively privileged university professors (XXX), yet has since shifted so far to the far-right that even other European far-right parties refuse to collaborate with it (YYY)."

**Author’s response**: We have cited the first statement using an academic source. The second is hard to quote with an academic source, as it is a factual statement that the AfD was expelled from the right-wing European faction ID in May 2024 and the ECR group (Meloni, Sweden Democrats) also refused them, which both have made statements about. We now mention this explicitly rather than citing an academic source for it.

**Editor’s request**: 3/It seems important to pay much closer attention to the information in the table notes for the regression tables: what is the procedure? (you say "xtreg fe" for Table 2, do the same for Table 1); what is the nature of the confidence-estimate statistics (standard deviation of the estimate? But there are negative figures... what does that mean?; please provide something simple); and finally, you refer to "average marginal effect," but you interpret them as odds ratios (less than 1 = negative effect); the very nature of the coefficients you provide in the tables must be clarified, otherwise we even question your interpretations.

**Author’s response**: I have added to the notes of Table 1: “Table shows odds ratios based on Stata xtlogit re procedure with robust standard errors”, also making clear what the confidence intervals are showing. The explanation you were looking for is already in the data and methods section: “Odds ratios can approach infinity for positive effects, but are bounded at 0 for negative effects, which makes positive and negative effects difficult to compare. We therefore illustrate all effects using average marginal effects plots, which show by how many percentage points an individual’s probability to support the AfD changes if an independent variable increases by one standard deviation.“ However, I now reiterate in the results section: „ Model 4 takes up all variables simultaneously. Keep in mind that Table 1 shows odds ratios, which can approach infinity for positive effects, but are bounded at 0 for negative effects, which makes positive and negative effects difficult to compare. Figure 2 therefore visualizes the effects of Model 4 by converting odds ratios into average marginal effects, showing how one standard deviation increase of each independent variable is related to the probability of AfD support, irrespective of changes in the other variables. While the tables thus show effects as odds ratios (whose lower bound is 0 and upper bound is infinity), Figure 2 converts this into average marginal effects, which shows effects measured as changes in the probability to support the AfD on a scale from 0-1.” I hope that by reiterating this in the results section, it is now clearer.
