# Supplementary material for: Supporters of Germany’s far-right AfD party are concerned about immigration rather than feeling deprived
Source: PLoS One. 2026 Jul 21;21(7):e0350403. doi: 10.1371/journal.pone.0350403 (PMC13387537; doi:10.1371/journal.pone.0350403)
Supplement: Peer review history

# Peer review: Decision Letter 0

**DOI:** 10.1371/journal.pone.0350403.r001

**Authors:** Bruno Ventelou

9 Jan 2026

PONE-D-25-58118 Supporters of Germany’s far-right AfD party are concerned about immigration rather than feeling deprived PLOS One

Dear Dr. Schröder,

Thank you for submitting your manuscript to PLOS ONE. After careful consideration, we feel that it has merit but does not fully meet PLOS ONE’s publication criteria as it currently stands. Therefore, we invite you to submit a revised version of the manuscript that addresses the points raised during the review process.

Please submit your revised manuscript by Feb 21 2026 11:59PM. If you will need more time than this to complete your revisions, please reply to this message or contact the journal office at plosone@plos.org. When you're ready to submit your revision, log on to https://www.editorialmanager.com/pone/ and select the 'Submissions Needing Revision' folder to locate your manuscript file.

Please include the following items when submitting your revised manuscript:

- A letter that responds to each point raised by the academic editor and reviewer(s). You should upload this letter as a separate file labeled 'Response to Reviewers'.
- A marked-up copy of your manuscript that highlights changes made to the original version. You should upload this as a separate file labeled 'Revised Manuscript with Track Changes'.
- An unmarked version of your revised paper without tracked changes. You should upload this as a separate file labeled 'Manuscript'.

If you would like to make changes to your financial disclosure, please include your updated statement in your cover letter. Guidelines for resubmitting your figure files are available below the reviewer comments at the end of this letter.

If applicable, we recommend that you deposit your laboratory protocols in protocols.io to enhance the reproducibility of your results. Protocols.io assigns your protocol its own identifier (DOI) so that it can be cited independently in the future. For instructions see: https://journals.plos.org/plosone/s/submission-guidelines#loc-laboratory-protocols. Additionally, PLOS ONE offers an option for publishing peer-reviewed Lab Protocol articles, which describe protocols hosted on protocols.io. Read more information on sharing protocols at https://plos.org/protocols?utm_medium=editorial-email&utm_source=authorletters&utm_campaign=protocols.

We look forward to receiving your revised manuscript.

Kind regards,

Bruno Ventelou

Academic Editor

PLOS One

Journal Requirements:

When submitting your revision, we need you to address these additional requirements.

1. Please ensure that your manuscript meets PLOS ONE's style requirements, including those for file naming. The PLOS ONE style templates can be found at

https://journals.plos.org/plosone/s/file?id=wjVg/PLOSOne_formatting_sample_main_body.pdf and

https://journals.plos.org/plosone/s/file?id=ba62/PLOSOne_formatting_sample_title_authors_affiliations.pdf

2. We are unable to open your Supporting Information file [File Name]. Please kindly revise as necessary and re-upload.

3. We noted in your submission details that a portion of your manuscript may have been presented or published elsewhere. “We have uploaded a previous version of this article on a preprint server:

https://www.ssoar.info/ssoar/handle/document/105248

The authors that were still part of the project at the time we have uploaded this have agreed to leave the project though." Please clarify whether this [conference proceeding or publication] was peer-reviewed and formally published. If this work was previously peer-reviewed and published, in the cover letter please provide the reason that this work does not constitute dual publication and should be included in the current manuscript.

4. Please note that your Data Availability Statement is currently can’t access to a direct link. If your manuscript is accepted for publication, you will be asked to provide these details on a very short timeline. We therefore suggest that you provide this information now, though we will not hold up the peer review process if you are unable.

5. If the reviewer comments include a recommendation to cite specific previously published works, please review and evaluate these publications to determine whether they are relevant and should be cited. There is no requirement to cite these works unless the editor has indicated otherwise.

Additional Editor Comments :

I am making a decision based on a single report, in order to avoid any further delays. I suggest you carefully follow the remarks of the referee. The point about panel data would really make a difference.

[Note: HTML markup is below. Please do not edit.]

Reviewers' comments:

Reviewer's Responses to Questions

**Comments to the Author**

1. Is the manuscript technically sound, and do the data support the conclusions?

The manuscript must describe a technically sound piece of scientific research with data that supports the conclusions. Experiments must have been conducted rigorously, with appropriate controls, replication, and sample sizes. The conclusions must be drawn appropriately based on the data presented.

Reviewer #1: Yes

**********

2. Has the statistical analysis been performed appropriately and rigorously?

Reviewer #1: Yes

**********

3. Have the authors made all data underlying the findings in their manuscript fully available?

The PLOS Data policy requires authors to make all data underlying the findings described in their manuscript fully available without restriction, with rare exception (please refer to the Data Availability Statement in the manuscript PDF file). The data should be provided as part of the manuscript or its supporting information, or deposited to a public repository. For example, in addition to summary statistics, the data points behind means, medians and variance measures should be available. If there are restrictions on publicly sharing data—e.g. participant privacy or use of data from a third party—those must be specified.

Reviewer #1: Yes

**********

4. Is the manuscript presented in an intelligible fashion and written in standard English?

PLOS ONE does not copyedit accepted manuscripts, so the language in submitted articles must be clear, correct, and unambiguous. Any typographical or grammatical errors should be corrected at revision, so please note any specific errors here.

Reviewer #1: Yes

**********

5. Review Comments to the Author

Please use the space provided to explain your answers to the questions above. You may also include additional comments for the author, including concerns about dual publication, research ethics, or publication ethics. (Please upload your review as an attachment if it exceeds 20,000 characters)

Reviewer #1: This is a nice empirical contribution to the literature on determinants of far right-wing party support in general and on support for the far-right Alternative for Germany in particular. The paper therefore provides new insights into a societally highly relevant and scientifically highly important topic. The authors make use of the GSOEP dataset which covers a large number of cases for every year between 2014 and 2023, which allows the authors to estimate if feeling deprived or concerns about immigration matter for considering choosing the AfD in an election. The authors, using sophisticated statistical models, show – convincingly – that concerns about immigration are driving the AfD vote, and not concerns feeling deprived. I like the paper a lot and think that the authors make an important contribution. However, there are a few aspects that the authors should consider when revising the paper.

First, the paper would benefit from a clear and distinct theory section. The current version of the manuscript provides a longer introduction in which the authors present a nicely literature review. On that basis, the authors present three hypotheses. From my perspective the paper would gain significantly in its scientific impact when the authors would present a solidly derived mechanism that shows why concerns about immigration should affect AfD support more than feeling deprived. The authors could refer here to discussions in their literature review and – in particular – on changes in the AfD programmatic profile (the AfD started as a party focusing on Euroscepticism, before concentrating on migration and immigration since 2015).

Secondly, I do not really understand why the authors do not make more use of the panel structure of the data the use. The main advantage of the GSOEP dataset is that it is a panel dataset and that it thus allows for drawing causal inferences, as the authors do in section 3.3 of their paper. However, the authors use the data to a large extent in their analysis as a simple survey and do not use the time-series character of the data adequately. For instance, the authors could argue in the theory section that changes in an individual’s position or attitude on immigration between the current and the previous should affect the probability to vote AfD. For example, if an individual becomes less in favour of immigration during the last year, she should be more likely to vote or support AfD.

Third, the online appendix provides several robustness checks in which the authors show that the results remain stable when controlling for selected demographic variables like net household income, sex, education, regional background, etc. I think that the authors could do a better job in not only connecting the selected control variables to standard theories of voting behaviour but could also include further variables that are explicitly relevant for analysing the support for radical right parties. For instance, the GSOEP data allows for coding the professional background of parties, and on that basis the authors could connect their study to the ongoing debate when and under which conditions workers increasingly switched to the AfD and if this social group did so in particular because of concerns about immigration. In this context, it would be extremely interesting to see when this pattern became observable during the time period under study (a recent analysis (https://link.springer.com/article/10.1007/s11615-025-00604-y) using GSOEP data shows that workers, even those with a former SPD identification, switched to the AfD in 2017, but were no AfD supporters in 2013 when the AfD highlighted their Eurosceptic positions and did less focus on immigration).

Along these lines, the authors could also consider doing the analysis on the basis of the pooled GSEP dataset covering all years from 2014 to 2023. On that basis, the authors could interact their substantive explanatory variables with dummy variables for each year to identify if the effects of the explanatory variables changed during the observation period.

**********

6. PLOS authors have the option to publish the peer review history of their article (what does this mean?). If published, this will include your full peer review and any attached files.

If you choose “no”, your identity will remain anonymous but your review may still be made public.

**Do you want your identity to be public for this peer review?** For information about this choice, including consent withdrawal, please see our Privacy Policy.

Reviewer #1: No

**********

[NOTE: If reviewer comments were submitted as an attachment file, they will be attached to this email and accessible via the submission site. Please log into your account, locate the manuscript record, and check for the action link "View Attachments". If this link does not appear, there are no attachment files.]

To ensure your figures meet our technical requirements, please review our figure guidelines: https://journals.plos.org/plosone/s/figures

You may also use PLOS’s free figure tool, NAAS, to help you prepare publication quality figures: https://journals.plos.org/plosone/s/figures#loc-tools-for-figure-preparation.

NAAS will assess whether your figures meet our technical requirements by comparing each figure against our figure specifications.

# Author comment: Author response to Decision Letter 1

**DOI:** 10.1371/journal.pone.0350403.r002

2 Mar 2026

Please see R&R document

Attachment Submitted filename: R&R document.docx

# Editor report: Decision Letter 1

**DOI:** 10.1371/journal.pone.0350403.r003

**Authors:** Bruno Ventelou

22 Mar 2026

PONE-D-25-58118R1 Supporters of Germany’s far-right AfD party are concerned about immigration rather than feeling deprived PLOS One

Dear Dr. Schröder,

Thank you for submitting your manuscript to PLOS ONE. After careful consideration, we feel that it has merit but does not fully meet PLOS ONE’s publication criteria as it currently stands. Therefore, we invite you to submit a revised version of the manuscript that addresses the points raised during the review process.

Please submit your revised manuscript by May 06 2026 11:59PM. If you will need more time than this to complete your revisions, please reply to this message or contact the journal office at plosone@plos.org. When you're ready to submit your revision, log on to https://www.editorialmanager.com/pone/ and select the 'Submissions Needing Revision' folder to locate your manuscript file.

Please include the following items when submitting your revised manuscript:

- A letter that responds to each point raised by the academic editor and reviewer(s). You should upload this letter as a separate file labeled 'Response to Reviewers'.
- A marked-up copy of your manuscript that highlights changes made to the original version. You should upload this as a separate file labeled 'Revised Manuscript with Track Changes'.
- An unmarked version of your revised paper without tracked changes. You should upload this as a separate file labeled 'Manuscript'.

If you would like to make changes to your financial disclosure, please include your updated statement in your cover letter. Guidelines for resubmitting your figure files are available below the reviewer comments at the end of this letter.

If applicable, we recommend that you deposit your laboratory protocols in protocols.io to enhance the reproducibility of your results. Protocols.io assigns your protocol its own identifier (DOI) so that it can be cited independently in the future. For instructions see: https://journals.plos.org/plosone/s/submission-guidelines#loc-laboratory-protocols. Additionally, PLOS ONE offers an option for publishing peer-reviewed Lab Protocol articles, which describe protocols hosted on protocols.io. Read more information on sharing protocols at https://plos.org/protocols?utm_medium=editorial-email&utm_source=authorletters&utm_campaign=protocols.

We look forward to receiving your revised manuscript.

Kind regards,

Bruno Ventelou

Academic Editor

PLOS One

**Journal Requirements:**

If the reviewer comments include a recommendation to cite specific previously published works, please review and evaluate these publications to determine whether they are relevant and should be cited. There is no requirement to cite these works unless the editor has indicated otherwise.

**Additional Editor Comments:**

Dear author, some remarks to improve the paper; before final acceptation

The abstract can be shortened; I would suggest to cancel “We show how an immigration-unconcerned but deprived “loser of modernization” still only has half the likelihood to support the AfD than an entirely non deprived “winner of modernization” who is concerned about immigration.” (And I would suppress “bivariate” which is not clear for a large audience; in place, I would suggest to use the term “direct” – for indicating another idea – see my point below).

I agree with you that, statistically, immigration concern is a better (direct) predictor of the AFD vote than economic insecurity. This is your main result and your point deserves to be published. However, some elements need to be acknowledged.

1/ You did not check whether economic insecurity was (previously) the underlying cause of immigration concern. To do this, structural modelling (multiple equation estimation) would be necessary. I'm not explicitly asking for it, but the lack of a genuine analysis of opinion formation on immigration must be acknowledged.

2/ I'm almost certain that, if you test, an interaction effect between immigration and deprivation (statistical effect of ‘immigration x deprivation’) on AFD-support, it would be significant. As an economist, I would be interested to see the result; this is another way to test that the formation of immigration concern and economic deprivation synergize (…or not! if I am wrong).

3/ Please reread the article. I think you're overestimating the result concerning deprivation (you can't claim that there's no effect of deprivation, or even a weak one (fixed effects model, model 2, Table 2: the effect is 1.32, p < 0.001, which is significant, even if it is smaller than that of the "cultural" variable)). Please correct the paper when you overstate the no-relationship.

The panel data analysis (fixed-effect) is crucial for your demonstration. However:

-Model 1 / 2 / 3 / 4 should be presented better (please, think to a table that give a recap’ on the assumptions for the four models). Then, could you have a large Table of results (estimated coefficients) with all the 4 models?

-I don’t understand why you need bootstrapping to generate sd? (is it the same in Table 1?)

-Comparisons between models can be tested formally (LR test); this is better than only considering models’ respective R2.

-This is very important in the panel data analysis to be more precise on the waves; What are the dates for the past values of immigration-concern and deprivation?

[Note: HTML markup is below. Please do not edit.]

[NOTE: If reviewer comments were submitted as an attachment file, they will be attached to this email and accessible via the submission site. Please log into your account, locate the manuscript record, and check for the action link "View Attachments". If this link does not appear, there are no attachment files.]

To ensure your figures meet our technical requirements, please review our figure guidelines: https://journals.plos.org/plosone/s/figures

You may also use PLOS’s free figure tool, NAAS, to help you prepare publication quality figures: https://journals.plos.org/plosone/s/figures#loc-tools-for-figure-preparation.

NAAS will assess whether your figures meet our technical requirements by comparing each figure against our figure specifications.

# Author comment: Author response to Decision Letter 2

**DOI:** 10.1371/journal.pone.0350403.r004

30 Mar 2026

Please see R&R document

Attachment Submitted filename: R&R_document_auresp_2.docx

# Editor report: Decision Letter 2

**DOI:** 10.1371/journal.pone.0350403.r005

**Authors:** Bruno Ventelou

27 Apr 2026

PONE-D-25-58118R2Supporters of Germany’s far-right AfD party are concerned about immigration rather than feeling deprivedPLOS One

Dear Dr. Schröder,

Thank you for submitting your manuscript to PLOS ONE. After careful consideration, we feel that it has merit but does not fully meet PLOS ONE’s publication criteria as it currently stands. Therefore, we invite you to submit a revised version of the manuscript that addresses the points raised during the review process. See my comments at the end of this letter. Please take the time to resubmit a carefully cleaned-up version.

Please submit your revised manuscript by Jun 11 2026 11:59PM. If you will need more time than this to complete your revisions, please reply to this message or contact the journal office at plosone@plos.org. When you're ready to submit your revision, log on to https://www.editorialmanager.com/pone/ and select the 'Submissions Needing Revision' folder to locate your manuscript file.

Please include the following items when submitting your revised manuscript:

- A letter that responds to each point raised by the academic editor and reviewer(s). You should upload this letter as a separate file labeled 'Response to Reviewers'.
- A marked-up copy of your manuscript that highlights changes made to the original version. You should upload this as a separate file labeled 'Revised Manuscript with Track Changes'.
- An unmarked version of your revised paper without tracked changes. You should upload this as a separate file labeled 'Manuscript'.

If you would like to make changes to your financial disclosure, please include your updated statement in your cover letter. Guidelines for resubmitting your figure files are available below the reviewer comments at the end of this letter.

If applicable, we recommend that you deposit your laboratory protocols in protocols.io to enhance the reproducibility of your results. Protocols.io assigns your protocol its own identifier (DOI) so that it can be cited independently in the future. For instructions see: https://journals.plos.org/plosone/s/submission-guidelines#loc-laboratory-protocols. Additionally, PLOS ONE offers an option for publishing peer-reviewed Lab Protocol articles, which describe protocols hosted on protocols.io. Read more information on sharing protocols at https://plos.org/protocols?utm_medium=editorial-email&utm_source=authorletters&utm_campaign=protocols.

As the corresponding author, your ORCID iD is verified in the submission system and will appear in the published article. PLOS supports the use of ORCID, and we encourage all coauthors to register for an ORCID iD and use it as well. Please encourage your coauthors to verify their ORCID iD within the submission system before final acceptance, as unverified ORCID iDs will not appear in the published article. *Only* the individual author can complete the verification step; PLOS staff *cannot* verify ORCID iDs on behalf of authors.

We look forward to receiving your revised manuscript.

Kind regards,

Bruno Ventelou

Academic Editor

PLOS One

**Journal Requirements:**

Please review your reference list to ensure that it is complete and correct. If you have cited papers that have been retracted, please include the rationale for doing so in the manuscript text, or remove these references and replace them with relevant current references. Any changes to the reference list should be mentioned in the rebuttal letter that accompanies your revised manuscript. If you need to cite a retracted article, indicate the article’s retracted status in the References list and also include a citation and full reference for the retraction notice.

**Additional Editor Comments:**

Dear author, I apreciate the revision2 ; I still have some few remarks: 1/ I am surprized that the study of the interaction effect is not made within the fixed-effect modelling (both variables are time-varying, this should be possible); 2/ this sentence would deserve a bibliographic references, for each of the (two) propositions: "Germany might be special, as the AfD emerged as a party of economically liberal and relatively privileged university professors (XXX), yet has since shifted so far to the far-right that even other European far-right parties refuse to collaborate with it (YYY)." 3/It seems important to pay much closer attention to the information in the table notes for the regression tables: what is the procedure? (you say "xtreg fe" for Table 2, do the same for Table 1); what is the nature of the confidence-estimate statistics (standard deviation of the estimate? But there are negative figures... what does that mean?; please provide something simple); and finally, you refer to "average marginal effect," but you interpret them as odds ratios (less than 1 = negative effect); the very nature of the coefficients you provide in the tables must be clarified, otherwise we even question your interpretations.

[Note: HTML markup is below. Please do not edit.]

[NOTE: If reviewer comments were submitted as an attachment file, they will be attached to this email and accessible via the submission site. Please log into your account, locate the manuscript record, and check for the action link "View Attachments". If this link does not appear, there are no attachment files.]

To ensure your figures meet our technical requirements, please review our figure guidelines: https://journals.plos.org/plosone/s/figures

You may also use PLOS’s free figure tool, NAAS, to help you prepare publication quality figures: https://journals.plos.org/plosone/s/figures#loc-tools-for-figure-preparation.

NAAS will assess whether your figures meet our technical requirements by comparing each figure against our figure specifications.

# Author comment: Author response to Decision Letter 3

**DOI:** 10.1371/journal.pone.0350403.r006

30 Apr 2026

Please see R&R document

Attachment Submitted filename: R&R_document_auresp_3.docx

# Editor report: Decision Letter 3

**DOI:** 10.1371/journal.pone.0350403.r007

**Authors:** Bruno Ventelou

13 May 2026

Supporters of Germany’s far-right AfD party are concerned about immigration rather than feeling deprived

PONE-D-25-58118R3

Dear Dr. Schröder,

We’re pleased to inform you that your manuscript has been judged scientifically suitable for publication and will be formally accepted for publication once it meets all outstanding technical requirements.

Within one week, you’ll receive an e-mail detailing the required amendments. When these have been addressed, you’ll receive a formal acceptance letter and your manuscript will be scheduled for publication.

An invoice will be generated when your article is formally accepted. Please note, if your institution has a publishing partnership with PLOS and your article meets the relevant criteria, all or part of your publication costs will be covered. Please make sure your user information is up-to-date by logging into Editorial Manager at Editorial Manager® and clicking the ‘Update My Information' link at the top of the page. For questions related to billing, please contact billing support.

If your institution or institutions have a press office, please notify them about your upcoming paper to help maximize its impact. If they’ll be preparing press materials, please inform our press team as soon as possible -- no later than 48 hours after receiving the formal acceptance. Your manuscript will remain under strict press embargo until 2 pm Eastern Time on the date of publication. For more information, please contact onepress@plos.org.

Kind regards,

Bruno Ventelou

Academic Editor

PLOS One

Additional Editor Comments (optional):

PLEASE ALSO INFORM THE READER ABOUT THE FIGURES IN TABLE 2 and TABLE 2 bis. Probably they are odds ratio...

Reviewers' comments:

# Editor report: Acceptance letter

**DOI:** 10.1371/journal.pone.0350403.r008

**Authors:** Bruno Ventelou

PONE-D-25-58118R3

PLOS One

Dear Dr. Schröder,

I'm pleased to inform you that your manuscript has been deemed suitable for publication in PLOS One. Congratulations! Your manuscript is now being handed over to our production team.

At this stage, our production department will prepare your paper for publication. This includes ensuring the following:

* All references, tables, and figures are properly cited

* All relevant supporting information is included in the manuscript submission,

* There are no issues that prevent the paper from being properly typeset

You will receive further instructions from the production team, including instructions on how to review your proof when it is ready. Please keep in mind that we are working through a large volume of accepted articles, so please give us a few days to review your paper and let you know the next and final steps.

Lastly, if your institution or institutions have a press office, please let them know about your upcoming paper now to help maximize its impact. If they'll be preparing press materials, please inform our press team within the next 48 hours. Your manuscript will remain under strict press embargo until 2 pm Eastern Time on the date of publication. For more information, please contact onepress@plos.org.

You will receive an invoice from PLOS for your publication fee after your manuscript has reached the completed accept phase. If you receive an email requesting payment before acceptance or for any other service, this may be a phishing scheme. Learn how to identify phishing emails and protect your accounts at https://explore.plos.org/phishing.

If we can help with anything else, please email us at customercare@plos.org.

Thank you for submitting your work to PLOS ONE and supporting open access.

Kind regards,

PLOS ONE Editorial Office Staff

on behalf of

Dr. Bruno Ventelou

Academic Editor

PLOS One
